# The relationship between immune cell infiltration and necroptosis gene expression in sepsis: an analysis using single-cell transcriptomic data

**DOI:** 10.3389/fcimb.2025.1618438

**Published:** 2025-08-11

**Authors:** Shouyi Wang

**Affiliations:** Department of Pediatrics, Zhongnan Hospital of Wuhan University, Wuhan, China

**Keywords:** sepsis, necroptosis, immune cell infiltration, single-cell RNA sequencing, bioinformatics

## Abstract

**Background:**

Sepsis is a life-threatening organ dysfunction caused by a dysregulated host response to infection. It remains a significant medical challenge due to its high mortality rates and requires a deeper understanding of its underlying mechanisms. This study aims to elucidate the differential expression of necroptosis-related genes in sepsis and their impact on immune characteristics.

**Methods:**

We obtained gene expression profiles and single-cell RNA sequencing data from the Gene Expression Omnibus (GEO) database. Differentially expressed genes (DEGs) were identified using the limma package, and functional enrichment analysis was performed using the clusterProfiler package for Gene Ontology (GO) and Kyoto Encyclopedia of Genes and Genomes (KEGG) pathways. Gene Set Variation Analysis (GSVA) and Gene Set Enrichment Analysis (GSEA) were conducted to explore pathway enrichments. Immune cell infiltration differences between sepsis (SE) and healthy control (HC) groups were quantified using the single-sample Gene Set Enrichment Analysis (ssGSEA) algorithm. Differential marker genes between SE and HC groups were identified by single-cell data analysis using the Seurat and SingleR packages.

**Results:**

Our results revealed 849 necroptosis-related DEGs, with 843 upregulated and 16 downregulated in the SE group. Least Absolute Shrinkage and Selection Operator (LASSO) regression identified 22 key DEGs, including *CTSS*, *MAPK8*, and *MPRIP*. Among these, 157 necroptosis-related DEGs were consistently identified between SE and HC groups. GO analysis indicated significant enrichment in biological processes such as the regulation of apoptotic signaling pathways and IκB kinase/NF-κB signaling. KEGG pathway analysis revealed involvement in necroptosis, apoptosis, and NOD-like receptor signaling pathways. GSVA demonstrated that Wnt signaling was upregulated in the SE group. Significant differences in immune cell infiltration were observed between sepsis and healthy control groups, particularly in activated B cells and CD4 T cells. Single-cell RNA sequencing identified 33,287 cells categorized into 26 clusters, with neutrophils predominating. Key necroptosis genes such as *CTSS*, *TXN*, *MYH9*, *FPR1*, *FMR1*, and *MPRIP* exhibited differential expression patterns across various immune cell types.

**Conclusions:**

Our integrated bioinformatics approach provides insights into the role of necroptosis-related genes in sepsis pathogenesis and their influence on immune responses. These findings improve our understanding of sepsis mechanisms and may guide future therapeutic strategies targeting necroptosis pathways.

## Introduction

Sepsis, a life-threatening organ dysfunction caused by a dysregulated host response to infection, remains a significant global health challenge. According to the Global Burden of Diseases report, sepsis affected approximately 49 million people worldwide in 2017, causing around 11 million deaths and accounting for nearly 20% of all global deaths (Rudd et al., 2020; Gavelli et al., 2021). The mortality rate of sepsis has gradually decreased with the timely use of antibiotics, fluid resuscitation, and supportive therapies for multiple organ dysfunction over the past two decades. However, there is still a significant mortality rate and considerable room for improvement. In addition to the high health-related burden, septic shock is one of the most expensive pathological conditions to treat. Its estimated annual healthcare cost is $24 billion (McBride et al., 2020). Current diagnostic and therapeutic approaches for sepsis have limitations. They cannot accurately predict outcomes or effectively target the underlying pathophysiological processes (Cecconi et al., 2018). Consequently, there is an urgent need for novel biomarkers and therapeutic targets to improve the diagnosis, prognosis, and treatment.

Necroptosis is a regulated form of necrotic cell death. It has emerged as a critical player in the pathogenesis of various inflammatory diseases, including sepsis (Linkermann and Green, 2014). Unlike apoptosis, necroptosis is characterized by cell membrane rupture and the release of damage-associated molecular patterns (DAMPs), which can exacerbate inflammation and tissue injury (Pasparakis and Vandenabeele, 2015). The key mediators of necroptosis, such as receptor-interacting protein kinase 3 (RIPK3) and mixed lineage kinase domain-like protein (MLKL), are upregulated in sepsis and contribute to organ damage and mortality (Newton et al., 2016). Inhibition of necroptosis in animal models of sepsis has been shown to reduce mortality and improve outcomes (Newton and Manning, 2016), suggesting that targeting necroptosis might be a promising therapeutic strategy for sepsis. However, the specific functions of necroptosis-related genes (NRGs) in the development and progression of sepsis remain unclear.

Immune system dysregulation plays a key role in the development of sepsis. During the initial phase of sepsis, the exaggerated inflammatory response triggers the recruitment of a large number of neutrophils, which play a crucial role in pathogenic bacterial clearance but also cause tissue damage (Ioannou et al., 2022). Damaged cells act as endogenous inflammatory inducers, thereby promoting the inflammatory response. Furthermore, dendritic cells (DCs) can activate toll-like receptors (TLRs) and produce excessive pro-inflammatory factors that amplify the immune response. These hyperactive immune responses ultimately trigger a cytokine storm, severely impairing normal immune function (Xiao et al., 2018). With the development of sepsis, the over-activation stage gradually transforms into the immune paralysis stage (also called immunosuppression) (Zhang et al., 2019; Forceville et al., 2021). Therefore, improving the immune microenvironment is crucial for treating septic patients. Immunotherapy holds broad potential for clinical application in sepsis. However, in sepsis, the relationship between immune cell infiltration characterization and necroptosis remains unknown.

Necroptosis plays a pivotal role in inflammation and cell death. Understanding how NRGs are expressed and function in sepsis could offer valuable insights into the disease’s pathophysiology. In this study, we investigated the differential expression of NRGs in sepsis and their impact on immune characteristics. We utilize gene expression profiles from peripheral blood cell samples of sepsis patients and healthy controls. Additionally, single-cell RNA sequencing data were used to identify differentially expressed genes (DEGs) and perform functional enrichment analysis as well as evaluate immune cell infiltration patterns.

By utilizing gene expression data from the GEO database, we performed differential gene expression analysis, immune cell infiltration analysis, and also analyzed single-cell RNA sequencing data. Using these bioinformatics methods, we identified 157 necroptosis-related DEGs, clarified their functions, and examined their links to immune cell infiltration in sepsis. The study has been published online in preprint form (Wang, 2024). Our findings provide new insights into the molecular mechanisms of sepsis and highlight potential biomarkers and therapeutic targets for sepsis, a devastating condition.

## Materials and methods

### Data acquisition

This study obtained gene expression data and single-cell transcriptomic data from the Gene Expression Omnibus (GEO) database. The data were accessed using the R package GEOquery. The bulk RNA sequencing data were sourced from GSE131761, which included 15 healthy controls (HC) and 114 sepsis (SE) patients, as well as GSE154918, comprising 26 HC and 79 SE patients (sample details are provided in **Supplementary Table 1**). After normalization and batch effect correction, these datasets were combined into a single expression matrix representing 41 HC and 193 SE subjects. Furthermore, single-cell RNA sequencing data were obtained from GSE167363. Sample details are provided in **Supplementary Table 1**. The data included peripheral blood mononuclear cells (PBMCs) from 2 HC and 5 SE donors. Additionally, the necroptosis-associated gene set consisting of 242 genes, was curated from GeneCards and literature mining of established markers (Xie et al., 2022).

### Screening of differentially expressed genes associated with necroptosis

Differentially expressed genes (DEGs) were identified by comparing the PBMCs between sepsis (SE, n = 193) and healthy control (HC, n = 41) cohorts. The initial screening applied thresholds of |log_2_FC| ≥ 1 (indicating an absolute fold-change ≥ 2) and an adjusted P-value < 0.05, with the P-values corrected using the Benjamini-Hochberg method. The significance of gene expression differences was determined by Wilcoxon rank-sum testing, while clinical correlations were evaluated by univariate Cox regression. Following this screening, gene filtering identified 157 necroptosis-associated DEGs from GeneCards data. This gene set was further refined through LASSO regression for feature selection. The results were visualized using volcano plots to illustrate expression differences and heatmaps to display DEG patterns. Both visualizations were generated with the heatmap R package.

### Gene Ontology function and Kyoto Encyclopedia of Genes and Genomes pathway enrichment analysis

Functional enrichment of necroptosis-associated differentially expressed genes (DEGs) was performed using comprehensive Gene Ontology (GO) analysis, including biological processes (BP), cellular components (CC), and molecular functions (MF). Moreover, KEGG pathway annotation was performed to identify relevant biological pathways. This analysis used the clusterProfiler package (version 4.0), considering statistical significance at an FDR-adjusted P value < 0.05.

### Gene set variation analysis and gene set enrichment analysis

Gene set variation and enrichment analyses were performed using different computational methods. Gene Set Variation Analysis (GSVA), implemented via the GSVA package (v1.46.0), used pre-filtered necroptosis-associated DEGs (|log_2_FC| > 0.1 and P < 0.05) on the c2.cp.kegg.v7.5.1.symbols.gmt gene set from MSigDB, generating a pathway activity matrix (samples × pathways). Meanwhile, Gene Set Enrichment Analysis (GSEA), conducted with clusterProfiler (v4.0), ranked genes according to their correlation with phenotype expression and evaluated them against the c2.cp.v7.5.1.symbols.gmt collection (MSigDB v7.5.1), using a significance threshold of FDR < 0.25, as defined by Subramanian et al.

### Immune characterization analysis

The ssGSEA algorithm was selected for this study because it effectively quantifies immune cell infiltration. It also analyzes phenotype correlations between SE and HC groups. This choice utilizes the standardized immune cell reference panel from Charoentong’s study (Charoentong et al., 2017). The method is further supported by proven utility in similar immunological contexts and validation in the literature (Chen et al., 2022; Zhou et al., 2023a). Subsequent analyses applied Spearman’s correlation to identify significant immune cell-DEG relationships, which were then visualized using hierarchical clustering heatmaps. Additionally, comparative boxplots stratifying samples by DEG expression tertiles revealed differential immune patterns and subtype-specific variations in the immune microenvironment.

### Single-cell data analysis

Single-cell RNA-seq analysis was performed using Seurat (v4.0) for integrated processing. Quality control retained cells with less than 20% mitochondrial gene content and 200 to 3,000 detected genes (nFeature_RNA), excluding doublets and damaged cells. Following this, data were normalized using LogNormalize, and scaling was applied to remove the effects of sequencing depth. After normalization and scaling, 3,000 variable features were identified using the variance-stabilizing transformation (vst) method. Dimensionality reduction used 17 principal components selected based on the ElbowPlot, and the results were visualized with t-SNE embedding.

### Single-cell clustering and annotation

Single-cell clustering and annotation were performed using Seurat v4.0 with graph-based clustering at a resolution of 0.5, controlling the granularity of the clusters; this process was performed using the FindNeighbors() and FindClusters() functions. Cell typing used the Blueprint/ENCODE reference atlas with SingleR v1.8.1. This approach enabled annotation of major immune lineages, including B cells, CD4^+^/CD8^+^ T cells, monocytes, natural killer (NK) cells, neutrophils, and hematopoietic stem cells (HSCs). Following clustering and annotation, marker genes were identified using Wilcoxon rank-sum testing through the FindAllMarkers function, applying thresholds of adjusted P < 0.01 and absolute log_2_ fold change (|log_2_FC|) > 0.5. The results were visualized using heatmaps to highlight gene expression patterns related to necroptosis.

### Statistical analysis

Statistical analyses employed different methods. For comparing two groups of continuous variables, data were first tested for normality. Normally distributed data were analyzed using Student’s t-test, while non-normal data were assessed with the Mann-Whitney U test. Significance was set at a false discovery rate (FDR)-adjusted P < 0.05. Concurrently, single-cell analysis was performed using Seurat v4.0, starting with LogNormalize and vst-based selection of 3,000 variable genes. Next, data were scaled and sequencing depth effects were regressed out. Dimensionality reduction was performed using principal component analysis (PCA), selecting 17 principal components based on the ElbowPlot. Graph-based clustering was performed using K-nearest neighbors in PCA space with the Euclidean metric and optimized by the Louvain algorithm (resolution = 0.5). Visualization was then achieved using t-SNE. Differential gene expression analysis was performed using the Wilcoxon rank-sum test.

## Results

### Differentially expressed necroptosis genes between HC and SE groups

**Figure 1:**
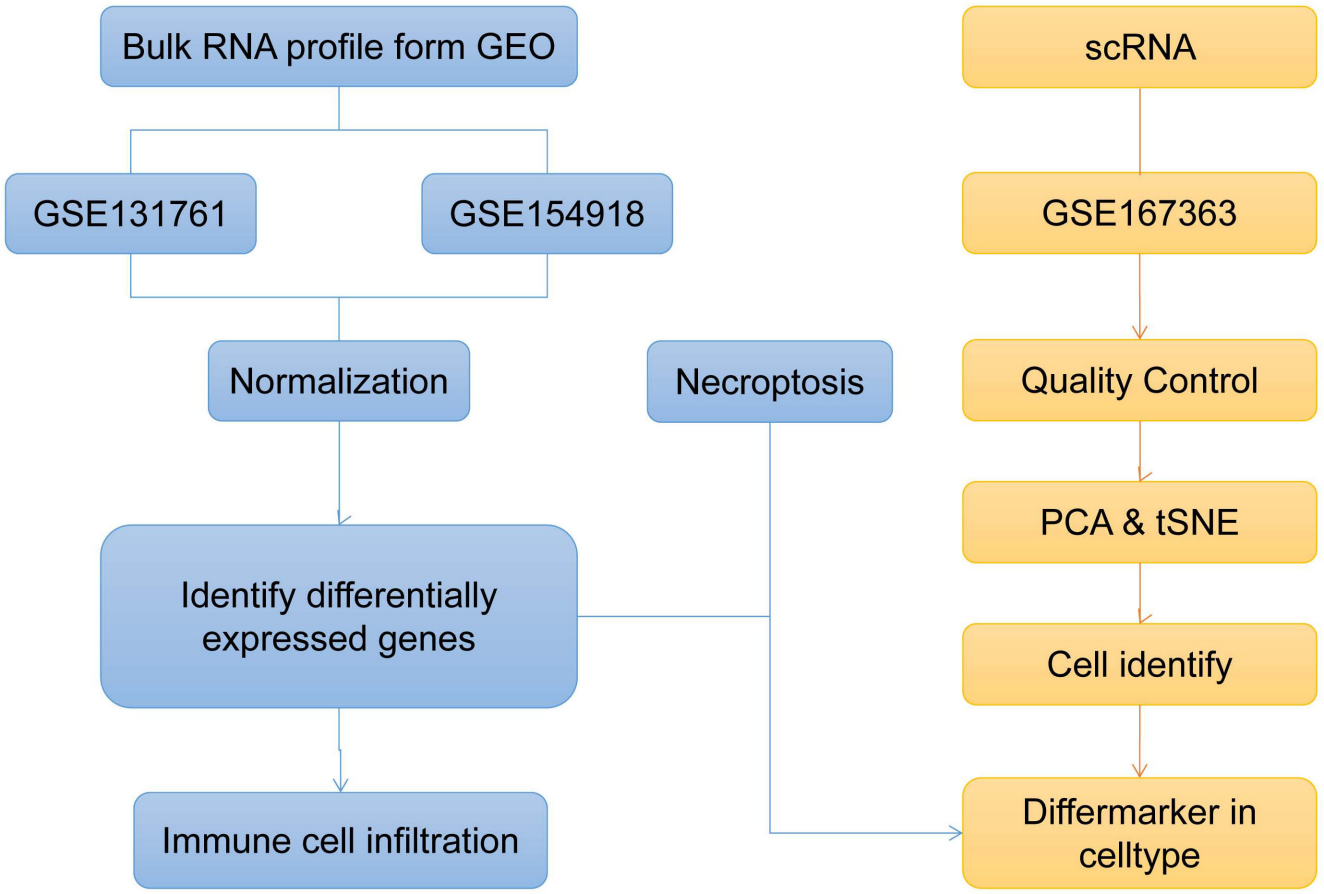
Bioinformatics method to explore the overall analysis flow chart of sepsis biological characteristics (t-SNE, t-distributed Stochastic Neighbor Embedding; PCA, Principal Component Analysis).

This investigation primarily focused on exploring the biological characteristics of SE using bioinformatics methodologies. The flowchart showing the analysis workflow is depicted in **Figure 1**. The samples were divided into two groups: the SE group and the HC group. Probe annotation and data normalization procedures were performed on the two GEO datasets. Boxplots were generated to visualize the data distribution before normalization (**Supplementary Figure S1A**) and after normalization (**Supplementary Figure S1B**), respectively.

Subsequently, the LIMMA package was used to analyze differences between two sample groups and to identify all necroptosis-related differential genes. A threshold of |log2(FC)| > 1 and p-value < 0.05 was set to identify necroptosis-related differentially expressed genes (DEGs), yielding 849 genes that met this criterion. Among these genes, 843 were upregulated, while 16 were downregulated.

The heatmap and volcano plot show differences in gene expression related to necroptosis. The volcano plot shows significant gene upregulation in sepsis (**Supplementary Figure S1C**), while the heat map reveals clear differences between the HC group and the SE group (**Supplementary Figure S1D**). These findings demonstrate that DEGs associated with necroptosis can effectively distinguish the SE group from the HC group.

### Differentiation of necroptosis in the SE and HC groups

**Figure 2:**
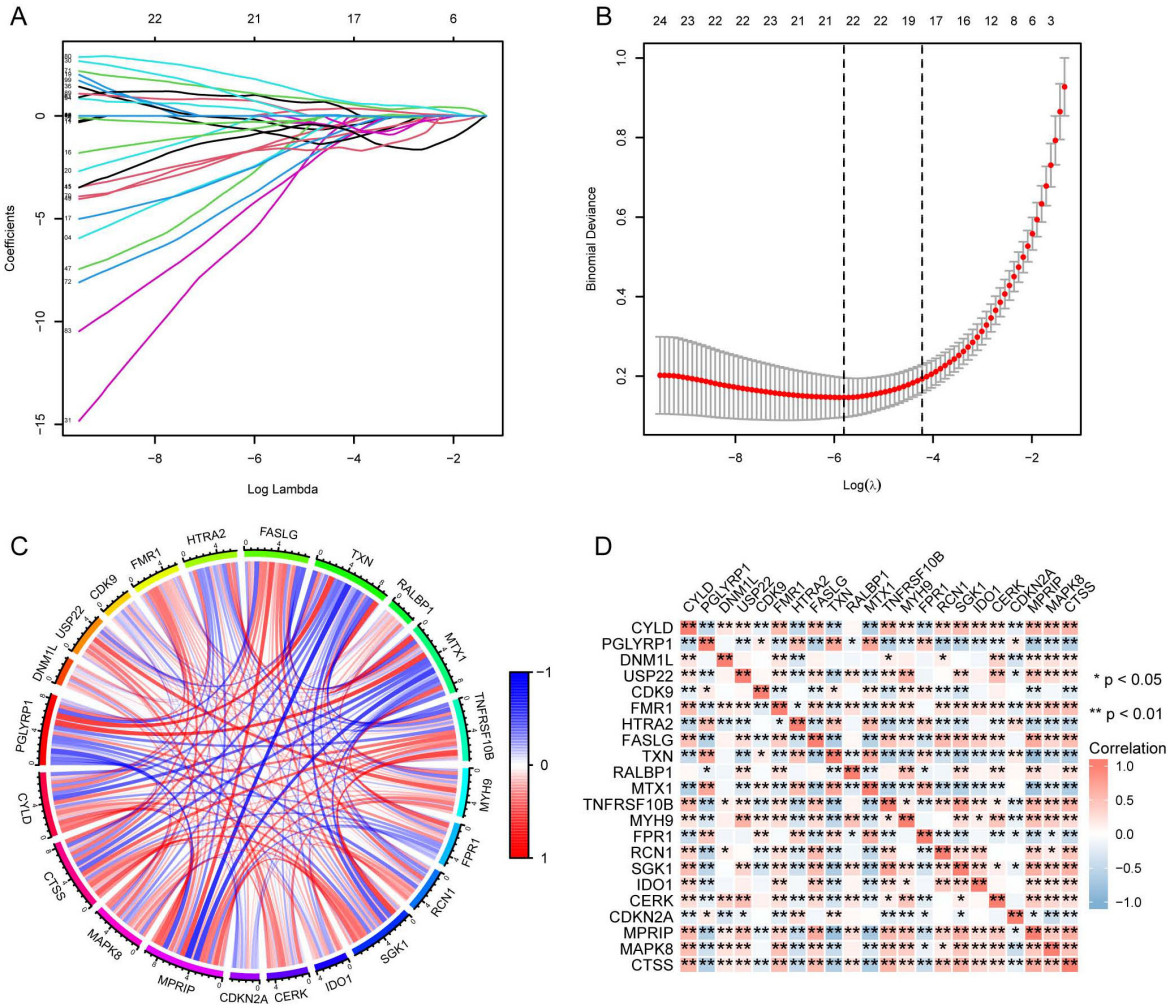
Visualization of DEGs based on LASSO regression using merged datasets. **(A)** Distribution of LASSO regression coefficients; **(B)** Determination of optimal parameters for LASSO regression; **(C)** Correlation plot between DEGs screened by LASSO regression, red for positive correlation and blue for negative correlation; **(D)** Correlation plot between DEGs screened by LASSO regression, with red representing positive correlation and blue representing negative correlation. (*Represents P ≤ 0.05, **Represents P ≤ 0.01; LASSO, least absolute shrinkage and selection operator; DEGs, differentially expressed genes).

**Figure 3:**
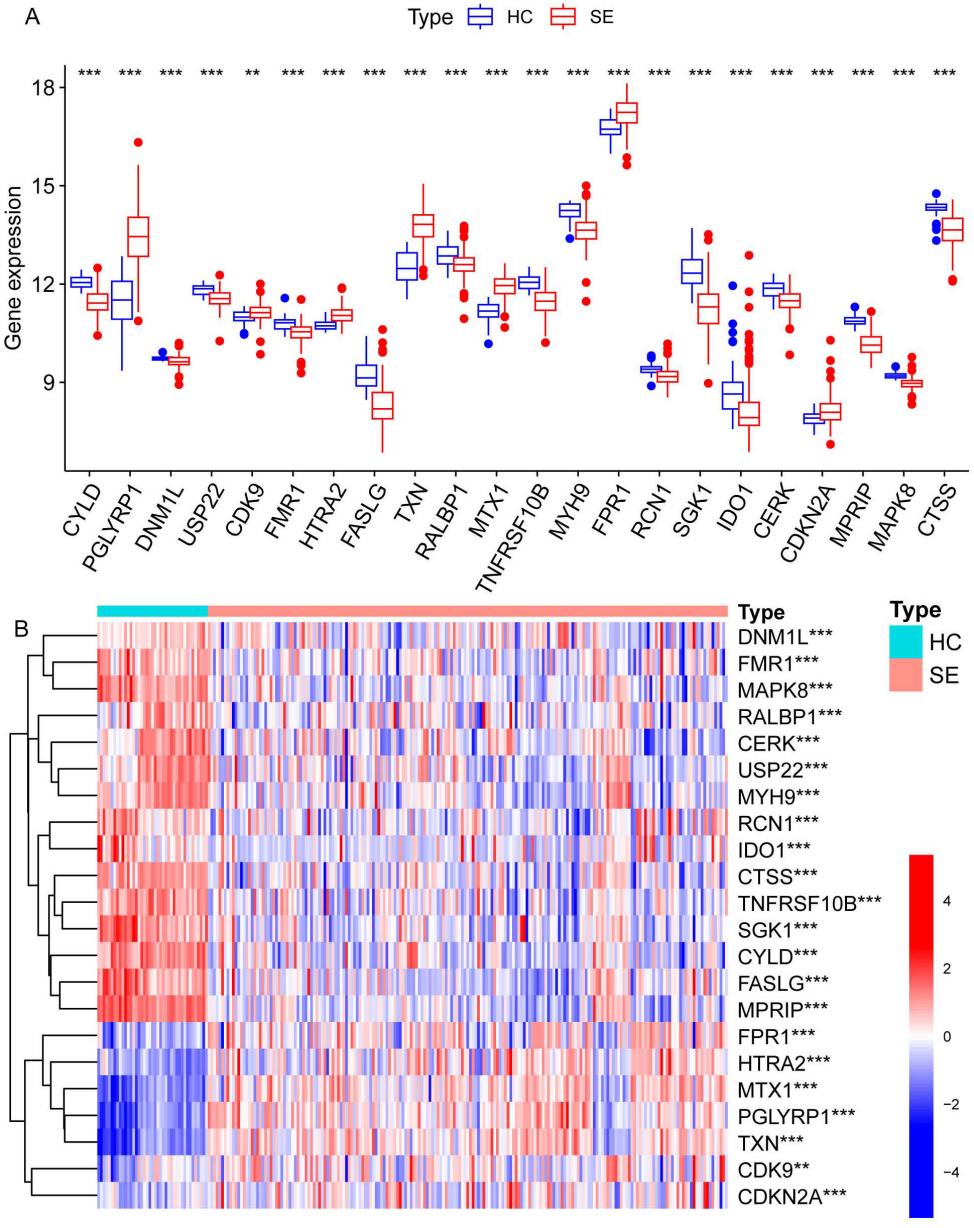
Visualization of expression differences in all necroptosis-related differential genes in the combined dataset. **(A)** The differentially expressed necroptosis genes screened by LASSO regression between the SE and HC groups, where red represents the SE group and blue represents the HC group. **(B)** The heatmap illustrates the differential gene expression of necroptosis screened by LASSO regression between the two groups, with red indicating high expression and blue indicating low expression. (**Represents P ≤ 0.01, ***Represents P ≤ 0.001; HC, Health control; SE, Sepsis; LASSO, least absolute shrinkage and selection operator).

We identified necroptosis-related genes differing between the SE and HC groups using the Wilcoxon rank sum test. Then, we further analyzed the differentially expressed necroptosis-related genes associated with SE using univariate Cox regression analysis. Using LASSO regression, we identified 22 significant necroptosis-related differential genes (**Figure 2A**). The optimal lambda value for the LASSO model was selected as shown in **Figure 2B**. Additionally, Based on LASSO regression, we examined interactions between DEGs using the Spearman correlation test. This analysis revealed a positive correlation between *TXN* (Thioredoxin) and *PGLYRP1* (Peptidoglycan Recognition Protein 1), and a negative correlation between *TXN* and *MPRIP* (Myosin Phosphatase Rho Interacting Protein) (**Figure 2C**). Further correlation analysis among *CTSS* (Cathepsin S), *MAPK8* (Mitogen-Activated Protein Kinase 8), *MPRIP*, and the other 21 genes revealed statistically significant associations (**Figure 2D**). To further investigate the expression differences of these 22 necroptosis genes, we generated box plots comparing the disease and control group (**Figure 3A**). The results showed statistically significant differences in expression levels. Furthermore, heat maps indicated a significantly increased expression of *USP22*, *CDK9*, *HTRA2*, *MTX1*, and *FPR1* in the SE group (**Figure 3B**). The full gene nomenclature is available in the “Gene Abbreviation Index” within the **Supplementary Materials**.

### GO biology functions, KEGG pathway, and GSVA

**Figure 4:**
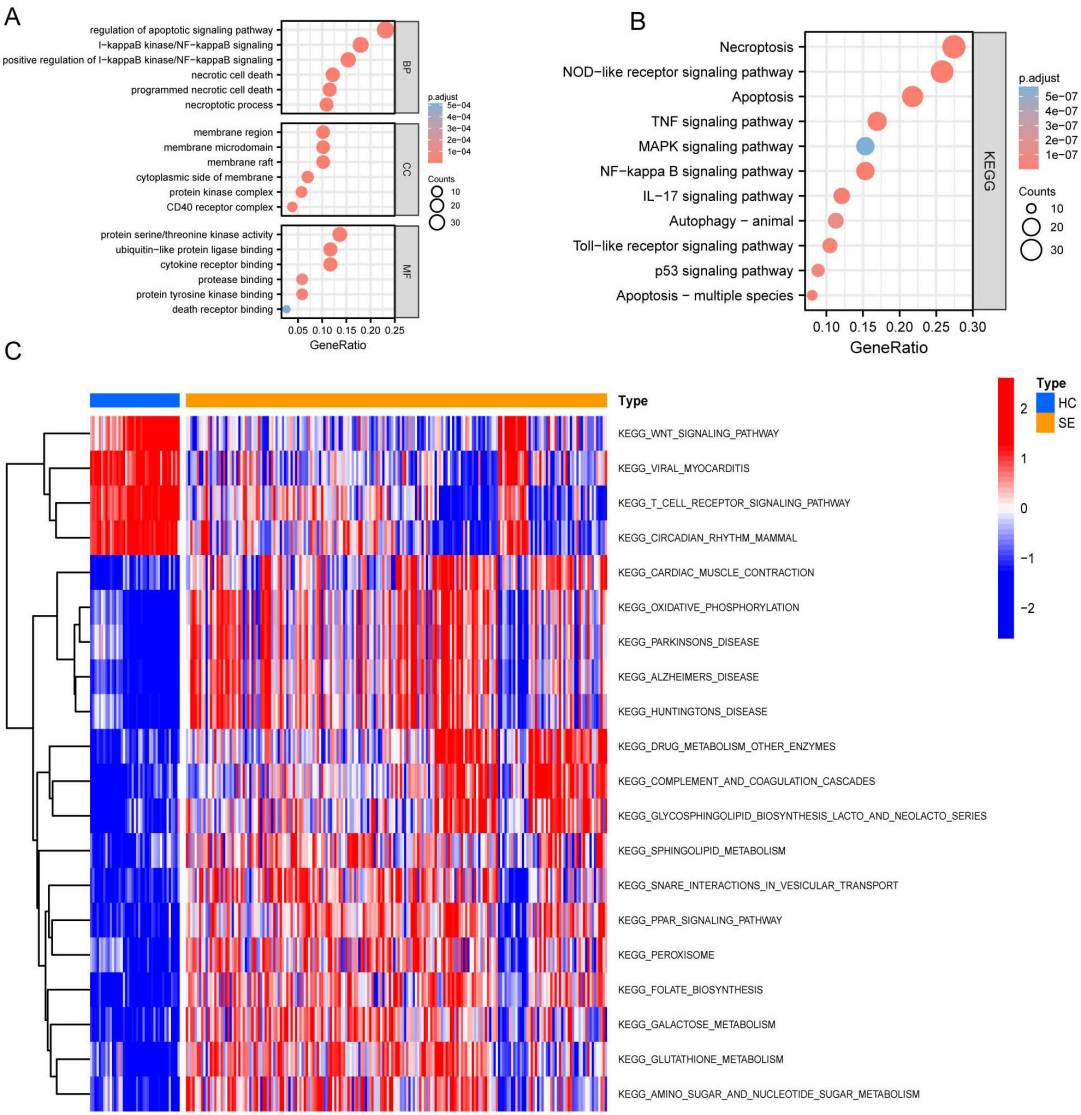
GO, KEGG, and GSVA based on GSE131761 and GSE154918 combined datasets. **(A)** Dot plot of GO enrichment analysis of necroptosis-associated DEGs in SE; **(B)** Dot plot of KEGG enrichment analysis of necroptosis associated DEGs in SE; **(C)** GSVA enrichment analysis heat map of DEGs in SE, red represents up-regulated pathways, and blue represents down-regulated pathways. (HC, Health control; SE, Sepsis; GO, Gene Ontology; KEGG, Kyoto Encyclopedia of Genes and Genomes; GSVA, Gene set variation analysis).

After identifying DEGs associated with necroptosis, we performed Gene Ontology (GO) and Kyoto Encyclopedia of Genes and Genomes (KEGG) analyses. These analyses aimed to explore pathway variations between the SE and HC groups. The results are shown in **Figures 4A, B**. GO analysis revealed significant enrichment in biological processes such as regulation and positive regulation of the apoptotic and IκB kinase*/*NF-κB signaling, as well as necrotic cell death and programmed necrotic cell death (**Figure 4A**). Additionally, enrichment was also observed in specific cellular components including the membrane region, membrane microdomain, membrane raft, cytoplasmic side of the membrane, and protein kinase complexes (**Figure 4A**). Furthermore, enrichment was noted in molecular functions such as protein serine/threonine kinase activity and binding activities to ubiquitin-like protein ligase binding, cytokine receptor binding, protease binding, and protein tyrosine kinase binding (**Figure 4A**).

Subsequently, we performed KEGG pathway enrichment analysis on the DEGs specifically related to necroptosis in SE. This analysis revealed significant enrichment in the following pathways: necroptosis pathway, NOD-like receptor signaling pathway, apoptosis pathway, TNF signaling pathway, MAPK signaling pathway, and several other pathways (P < 0.05) (**Figure 4B**).

Further Gene Set Variation Analysis (GSVA) (**Figure 4C**) shows significant differential gene expression in the oxidative phosphorylation and drug metabolism pathways involving other enzymes when comparing the HC group to the SE group. Specifically, a notable down-regulation is observed in the glycosphingolipid biosynthesis lacto and neolacto series pathways, as well as in the sphingolipid metabolism pathways. Conversely, there is a general upregulation in the pathways related to viral myocarditis, T cell receptor signaling pathway, circadian rhythm pathways in mammals, and the Wnt signaling pathway.

### Gene set enrichment analysis

**Figure 5:**
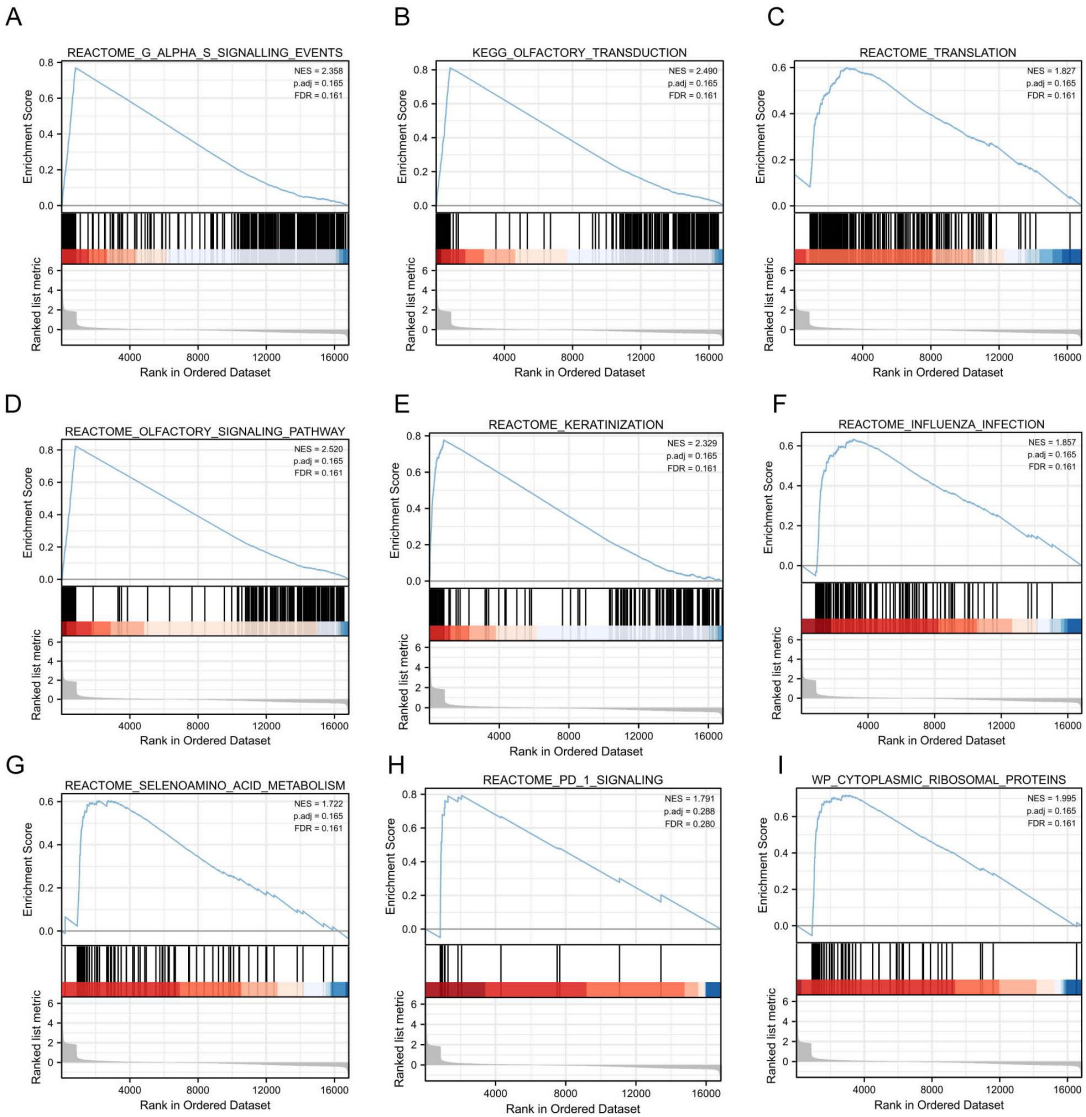
GSEA based on GSE131761 and GSE154918 combined datasets. **(A–I)**, Furthermore, the GSEA demonstrated that the differentially expressed genes in the pooled datasets of GSE131761 and GSE154918 were primarily enriched in the REACTOME_G_ALPHA_S_SIGNALLING_EVENTS **(A)**, KEGG_OLFACTORY_TRANSDUCTION **(B)**, REACTOME_TRANSLATION **(C)**, REACTOME_OLFACTORY_SIGNALING_PATHWAY **(D)**, REACTOME_KERATINIZATION **(E)**, REACTOME_INFLUENZA_INFECTIO **(F)**, REACTOME_SELENOAMINO_ACID_METABOLISM **(G)**, REACTOME_PD_1_SIGNALING **(H)**, WP_CYTOPLASMIC_RIBOSOMAL_PROTEINS **(I)**. (Satisfying the False discovery rate (FDR) < 0.25 and p < 0.05 were significant enrichments GSEA, Gene Set Enrichment Analysis).

To investigate the impact of gene expression levels on SE, we performed the Gene Set Enrichment Analysis (GSEA). We examined the relationship between gene expression and SE-associated biological pathways using combined datasets from GSE131761 and GSE154918. GSEA results showed significant gene enrichment in these categories: G alpha (s) signaling events (A), olfactory transduction (B), translation (C), olfactory signaling pathway (D), keratinization (E), influenza infection (F), selenoamino acid metabolism (G), PD-1 signaling (H), and cytoplasmic ribosomal proteins (I). These findings suggest significant changes in key biological functions and signaling pathways (**Figures 5A–I**).

### Immunological characterization among SE-related molecular subtypes

**Figure 6:**
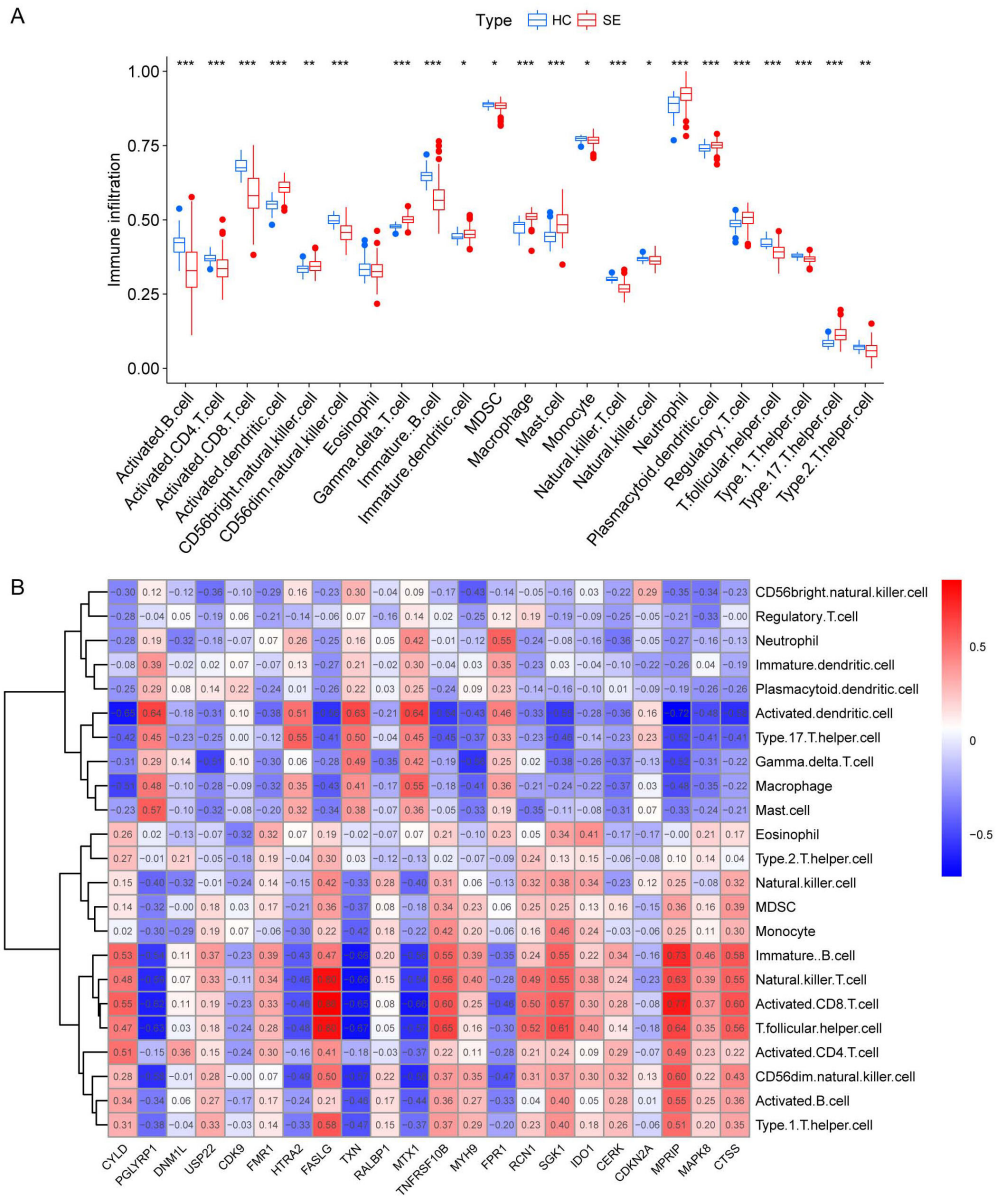
Immunological characterization between SE and HC based on pooled datasets. **(A)** A boxplot was generated to illustrate the variation in immune infiltration between the SE group (depicted in red) and the control group (depicted in blue). (*indicates P ≤ 0.05, **indicates P ≤ 0.01, ***indicates P ≤ 0.001). **(B)** Correlation heat map between immune cells and necroptosis-associated DEGs. The red color in the heat map indicates a positive correlation between the gene expression and the corresponding immune cell infiltration. The blue color indicates that the gene expression is negatively correlated with the corresponding immune cell infiltration. (HC, Health control; SE, Sepsis; DEGs, differentially expressed genes).

**Figure 7:**
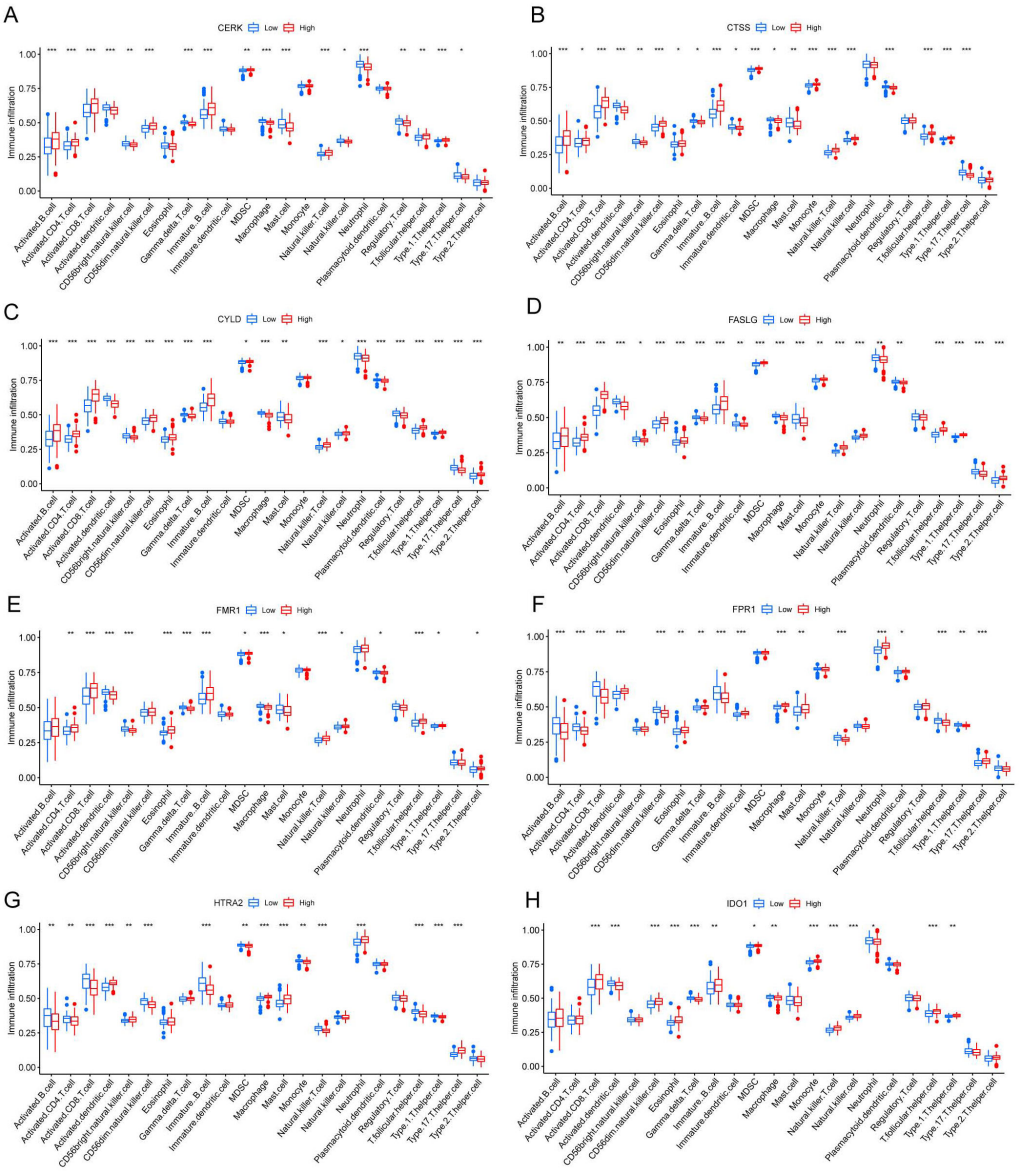
Correlation between different levels of necroptotic DEGs and immune infiltration. **(A-H)** CERK **(A)**, CTSS **(B)**, CYLD **(C)**, FASLG **(D)**, FMR1 **(E)**, FPR1 **(F)**, HTRA2 **(G)**, IDO1 **(H)** Correlation between different groupings of differentially expressed genes associated with necroptosis and immune infiltration. The blue color represents the low expression group, and the red color represents the high expression group. (*indicates P ≤ 0.05, **indicates P ≤ 0.01, ***indicates P ≤ 0.001); DEGs, differentially expressed genes).

We investigated the variations in immunological signatures between SE and HC in the pooled dataset. To quantify the disparities in immune cell infiltration, we employed the ssGSEA method. The findings, presented in **Figure 6A**, revealed significant differences in the infiltration of immune cells, including activated B cells, activated CD4^+^ T cells, activated CD8^+^ T cells, CD56^bright^ natural killer cells and gamma-delta T cells, between SE and HC. Subsequently, we conducted an analysis and mapping of the correlation between immune cells and necroptosis-related DEGs in sepsis samples. We observed a positive correlation between immature B cells, natural killer T cells, activated CD8^+^ T cells, T follicular helper cells (Tfh), activated CD4^+^ T cells, CD56^dim^ natural killer cells, activated B cells, type 1 T helper cells and other immune cells with *MPRIP*, *MAPK8*, and *CTSS*. Conversely, *MPRIP*, *MAPK8*, and *CTSS* showed a negative correlation with CD56^bright^ natural killer cells, regulatory T cells (Treg), neutrophils, immature dendritic cells, plasmacytoid dendritic cells, activated dendritic cells, type 17 T helper cells, gamma-delta T cells, macrophages, and mast cell (**Figure 6B**). To explore the relationship between the expression of individual DEGs and immune cells, we compared the differences in immune cell infiltration between high and low expression groups of each DEG. Our results demonstrated a connection between different expression groups of DEGs and the infiltration of immune cells. Notable distinctions were observed between the *CTSS* high and low expression groups regarding various immune cell populations (**Figure 7**). These included activated dendritic cells, CD56^bright^ natural killer cells, CD56^dim^ natural killer cells, eosinophils, gamma-delta T cells, immature B cells, immature dendritic cells, myeloid-derived suppressor cells (MDSC), macrophages, mast cells, monocytes, natural killer T cells, natural killer cells, plasmacytoid dendritic cells, T follicular helper cells, type 1 T helper cells and type 17 T helper cells.

### Single-cell heterogeneity in peripheral blood mononuclear cells

Seven single-cell sequencing samples were extracted from the single-cell dataset and analyzed. The sample information is presented in **Supplementary Table 1** (see **Supplementary Material**). In accordance with the quality control (QC) standards outlined in the methodology, a total of 33,287 cells were obtained. The violin plot shows the number of genes in each sample before and after QC (**Supplementary Figures S2A, G**). It was observed that the distribution of gene counts across samples became more consistent after QC filtering. A comparison between the SE group and HC group revealed a higher level of gene consistency between these groups (**Supplementary Figures S2D, J**). The distribution of nCount for each sample is shown before (**Supplementary Figures S2B, E**) and after QC procedures (**Supplementary Figures S2H, K**). Additionally, cells with high mitochondrial ratios were filtered before (**Supplementary Figures S2C, F**) and after QC (**Supplementary Figures S2I, L**). After applying QC measures, high-quality data were obtained for subsequent analysis.

**Figure 8:**
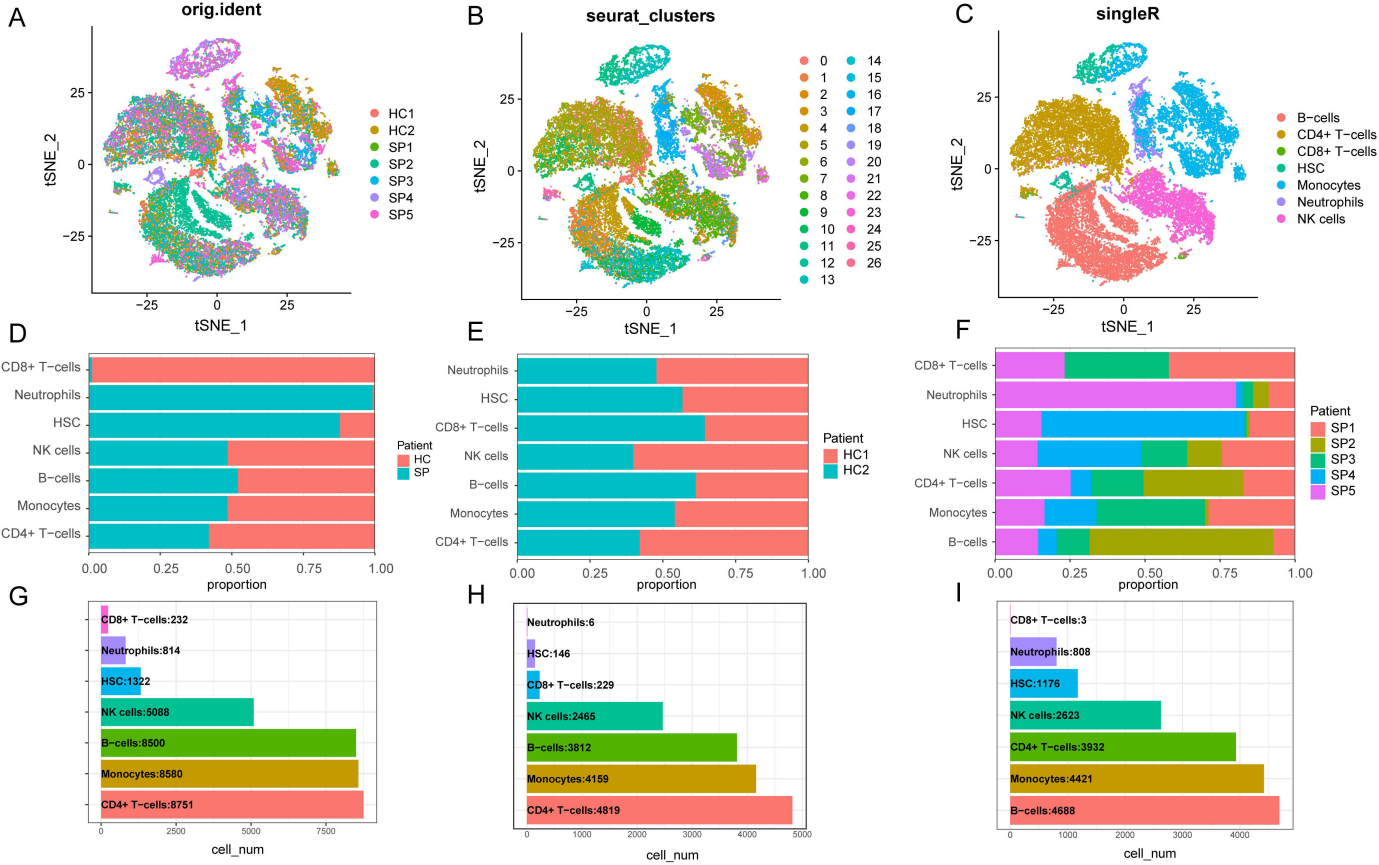
Dimensionality reduction clustering and cell annotation of single-cell data. **(A)** Distribution of t-SNE between different samples. **(B)** t-SNE distribution of different cell clusters, with different colors used to label clusters. UMI (unique molecular identifiers). **(C)** t-SNE distribution of cell types after Single R annotation. **(D)** The proportion of cells in each cell cluster in the control group and the disease group. **(E)** The proportion of cells from each type of cell cluster in the sample in the healthy control group. **(F)** The proportion of cells from various cell clusters in the patient sample in the disease group. **(G)** The number of cells of each type of cell cluster in the samples of the healthy control group and the SP group. **(H)** The number of cells of each type of cell cluster in the sample in the healthy control group. **(I)** The number of cells of each type of cell cluster in the SP group. (t-SNE, t-distributed Stochastic Neighbor Embedding. HC, Health control; SP, Sepsis Patients).

The “vst” method was implemented using the “Find Variable Features” function to detect 3000 variable traits within the dataset. The top 10 genes identified were *SERPINB2*, *CCL2*, *LCN2*, *PPBP*, *C1QB*, *PF4*, *C1QA*, *JCHAIN*, *MT2A*, and *S100A8* (**Supplementary Figures S3A, B**). After PCA dimensionality reduction analysis, the samples showed more consistent distribution (**Supplementary Figure S3C**), as indicated by the principal components (PCs) distribution plot and standard deviation. When the PC value reached 17, the curve distribution became relatively uniform. Therefore, the first 17 PCs were selected for further analysis (**Supplementary Figure S3D**). We visualized the cell distribution of seven samples using t-SNE dimensionality reduction, which revealed a more consistent cell distribution area across the samples (**Figure 8A**). Subsequently, 33,287 cells were classified into 26 distinct clusters (**Figure 8B**). Cell type identification using SingleR resulted in seven cell types: B cells, CD4^+^ T cells, CD8^+^ T cells, HSC, monocytes, neutrophils and NK cells (**Figure 8C**).

**Figure 9:**
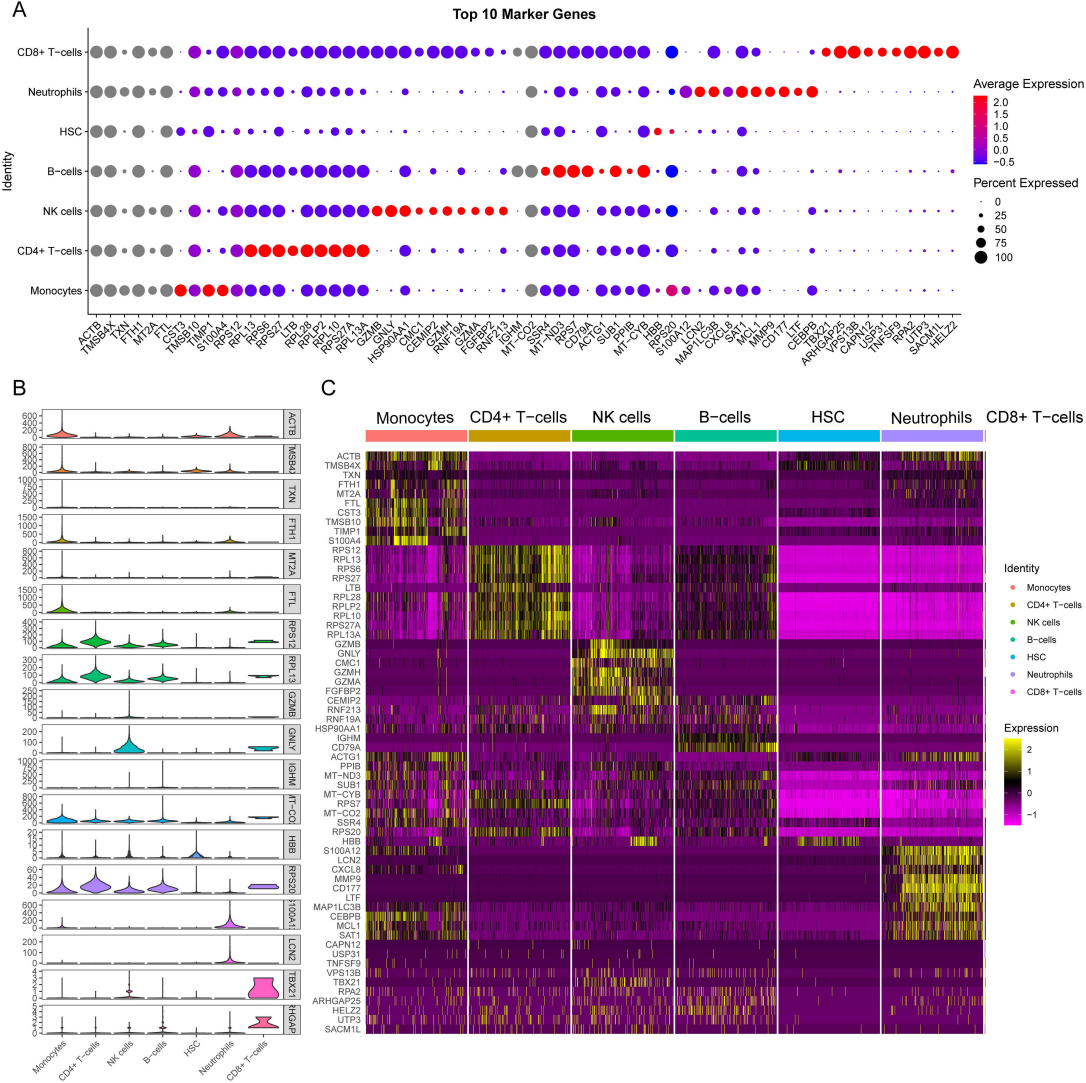
Single-cell sequencing analysis to explore the cell localization of marker genes. **(A)** The dot plot shows the top 10 differential genes for each cell type. The size of dots represents percent of cells expressing selected, and the color of dots represents average expression. The darker the color, the higher the average expression. **(B)** Violin plot depicts the top 2 differential genes for each cell type. **(C)** the heat map shows the expression of the top 10 differentially expressed genes in each cell type. Yellow represents high expression, and purple represents low expression.

We presented the number of cells in each class and the proportion of each cell type in each sample. The results showed that neutrophils had the highest proportion in the disease group, whereas CD8^+^ T cells were predominant in the control group (**Figure 8D**). There was minimal difference observed in the cellular distribution between the two samples within the healthy control group (**Figure 8E**). Among the five samples within the disease group, the sample labeled SP5 showed a greater proportion of neutrophils, while SP2 showed a higher proportion of B cells (**Figure 8F**). Across all samples, CD4^+^ T cells constituted the largest proportion relative to other cell types, whereas CD8^+^ T cells accounted for the smallest proportion among the cell types analyzed (**Figure 8G**). To visually represent these findings, we created a bubble map (**Figure 9A**) and a heatmap (**Figure 9C**) using the top 10 marker genes from various cell types. Notably, the 17 genes *S100A12*, *LCN2*, *MAP1LC3B*, *CXCL8*, *SAT1*, *MCL1*, *MMP9*, *CD177*, *LTF*, *CEBPB*, *RPL13*, *RPS6*, *RPS27*, *LTB*, *RPL28*, *RPLP2*, and *RPL10* showed specific expression in neutrophils. In addition, genes *RPS27A* and *RPL13A* exhibited specific and elevated expression in CD4^+^ T cells (**Figure 9C**). The top two marker genes were depicted in violin plots, highlighting their role as key marker genes among cells (**Figure 9B**).

**Figure 10:**
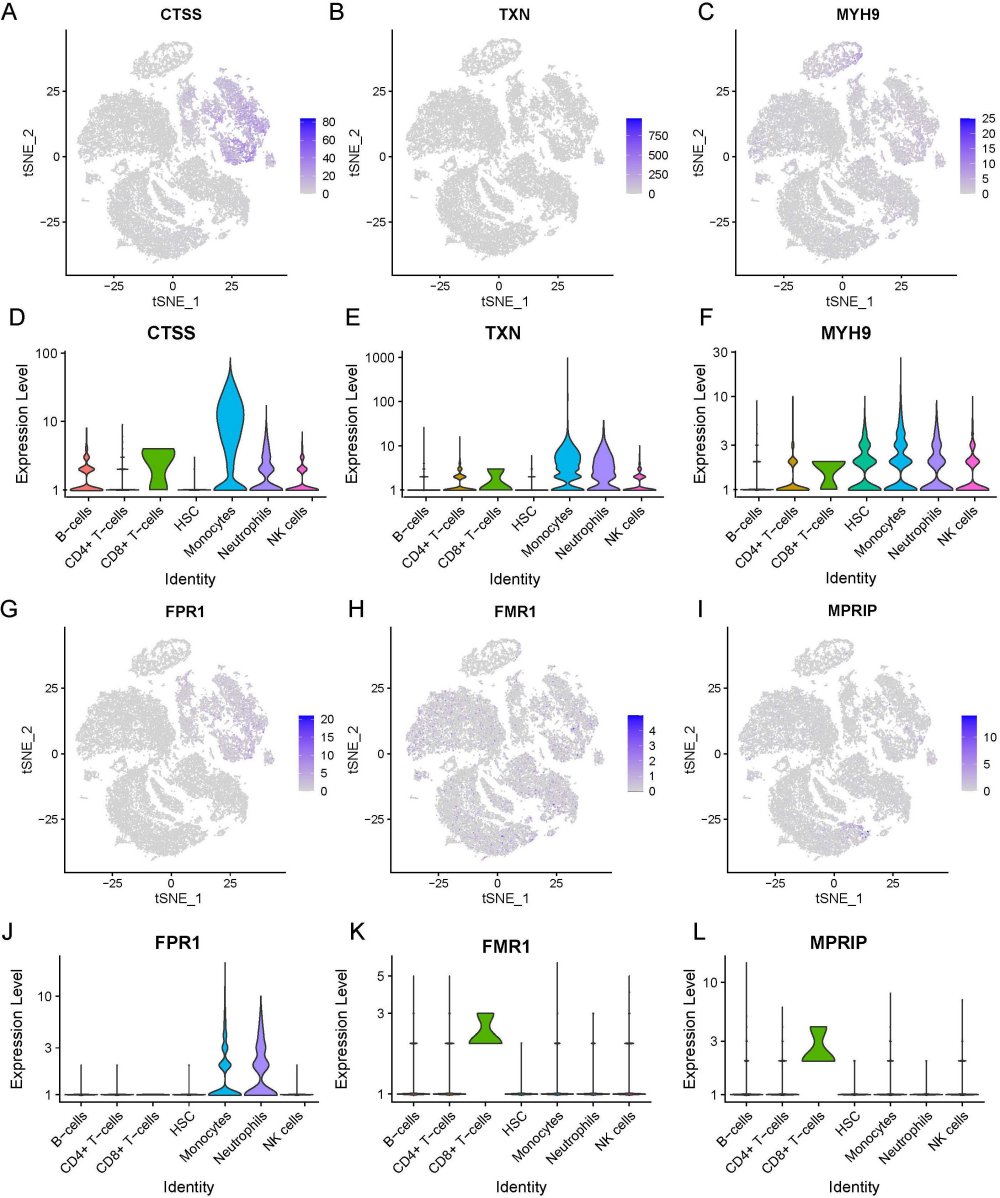
Expression of necrotizing apoptosis-related genes and immune cell differential genes in sepsis based on the combined dataset Lasso regression at the single-cell level. **(A-F)** t-SNE projection of CTSS **(A)**, TXN **(B)**, MYH9 **(C)**, FPR1 **(D)**, FMR1 **(E)** and MPRIP **(F)**. **(G-L)** Violin plot depicts CTSS **(A)**, TXN **(B)**, MYH9 **(C)**, FPR1 **(D)**, FMR1 **(E)** and MPRIP **(F)**. (LASSO, least absolute shrinkage and selection operator; t-SNE, t-distributed Stochastic Neighbor Embedding).

Six necroptosis-related genes (*CTSS, TXN, MYH9, FPR1, FMR1*, and *MPRIP*) exhibiting differential expression in immune cells were identified. This was achieved through an intersection analysis of necroptosis-associated genes and immune cell DEGs (**Supplementary Table 2**). We further compared the expression levels of these genes between HC and SE groups across major immune cell subsets, including B cells, CD4^+^ T cells, CD8^+^ T cells, monocytes, neutrophils, and NK cells. The analysis was visualized via t-SNE and violin plots (**Figures 10A-L**). The findings were as follows: (1) *CTSS, TXN, MYH9*, and *FPR1* showed significantly elevated expression in neutrophils and monocytes of SE patients compared to HC (P < 0.01); (2) *FMR1* and *MPRIP* demonstrated markedly higher expression in CD8^+^ T cells of SE patients (P < 0.05). Taken together, these results indicate that necroptosis-related gene upregulation in sepsis is cell type-specific, with neutrophils and CD8^+^ T cells showing the most pronounced changes. This highlights the critical role of necroptosis gene activation specific to immune cell subsets in sepsis pathogenesis.

## Discussion

Sepsis is a life-threatening condition characterized by a dysregulated host response to infection that leads to organ dysfunction and high mortality rates (Singer et al., 2016). Despite a 52.8% decline in mortality from 1990 to 2017, sepsis still impacted 48.9 million people in 2017 alone. It resulted in 11.0 million lives annually; this accounts for 19.7% of global deaths (Rudd et al., 2020). This burden is magnified in pediatric populations, where sepsis drives approximately 33% of fatalities in the Pediatric Intensive Care Unit (PICU) in the United States, with an approximate 25% mortality rate (Weiss et al., 2015). Furthermore, refractory septic shock is associated with a 34% mortality rate (Weiss et al., 2017). Importantly, microbial invasion initiates pathological cascades that involve anti-inflammatory cytokines become central drivers of immune failure.

The disease process develops through interactions between pathogens and the host, which disrupt inflammatory balance. Following microbial invasion, anti-inflammatory mediators such as IL-10 paradoxically amplify immunosuppression. Elevated IL-10 levels correlate with the severity of septic shock and dysfunctional pro-inflammatory responses in critically ill patients (Ng et al., 2003). IL-10 acts as a potent suppressor of cytokine synthesis in T cells and macrophages. However, it also exacerbates immunosuppressive effects, contributing to immune dysfunction. Within this regulatory axis, neutrophils are the primary infiltrating cells that combat pathogens like Klebsiella pneumoniae (KPn) and normally restore tissue homeostasis by clearing apoptotic cells via efferocytosis. However, KPn infection subverts this process (Jondle et al., 2018) through the concerted activation of neutrophil necroptosis machinery—a regulated form of inflammatory cell death—along with concurrent suppression of apoptosis-associated phosphatidylserine (PS) externalization. Additionally, KPn infection enhances flippase activity, which anchors PS to the plasma membrane inner leaflet. These combined effects culminate in impaired macrophage efferocytosis of infected neutrophils. This coordinated dysregulation of cell death pathways further interacts with IL-27-mediated metabolic reprogramming deficits, together exacerbating the pathological cascade. Neonatal models show that IL-27 deficiency improves bacterial clearance, reduces inflammation, and enhances survival during E. coli infection (Povroznik et al., 2025). The KPn pneumonia model further corroborates that targeting the necroptosis pathway (via RIPK1/RIPK3 inhibitors) not only restores efferocytic capacity but also significantly improves disease outcomes (Jondle et al., 2018). Collectively, these findings show that abnormal reprogramming of immune cell death pathways works together with imbalances in the cytokine network. This synergy drives the progression of organ failure.

These pathological processes converge on a critical bottleneck. Although sepsis is recognized as a dysregulated host response, the specific mechanisms in different cell types that cause immune dysfunction remain unclear. Necroptosis, in particular, represents a key process that amplifies tissue damage while simultaneously reprogramming immune cell functionality in a lineage-dependent manner. Necroptosis emerges as a critical bottleneck that exacerbates tissue damage and reprograms immune cell functionality in a lineage-dependent manner. This phenomenon is vividly exemplified by the death modality switching in neutrophils during Klebsiella pneumoniae (KPn) infection. In this context, apoptosis, which should routinely occur, is replaced by necroptosis, compromising efferocytosis and driving persistent inflammation. To address this, our study uses multi-modal transcriptomics to investigate how necroptosis-associated genes regulate immune subpopulation dysregulation, spatial immunometabolism, and clinical heterogeneity in sepsis.

Necroptosis is a form of programmed cell death distinct from apoptosis. It has been implicated in various inflammatory diseases and is thought to play a critical role in the pathogenesis of sepsis (Linkermann and Green, 2014). Necroptosis should be considered as an early surge of cell death that could lead to acute or sustained inflammation. Consequently, targeting the molecular mediators of necroptosis represents a promising therapeutic direction for sepsis (Qu et al., 2022). Previous bioinformatics analyses have demonstrated a correlation between changes in immune cell infiltration and sepsis (Xu et al., 2020). However, despite the known roles of necroptosis and immune infiltration in sepsis, few bioinformatics studies have specifically explored the relationship between necroptosis and sepsis.

This study focuses on the expression of necroptosis-related genes in sepsis and their immunological characteristics, aiming to provide new insights into the disease’s pathogenesis and potential therapeutic targets. By integrating multiple bioinformatics approaches, this study identifies differentially expressed necroptosis-related genes and their associated pathways. These findings highlight the roles of these genes in the pathogenesis and immune modulation of sepsis. Functional enrichment analyses, including GO and KEGG, reveal key biological pathways involved in sepsis. Moreover, GSVA and GSEA provide a comprehensive view of pathway alterations between sepsis patients and healthy controls. To further explore these findings at a finer resolution, the study’s single-cell transcriptomic analysis elucidates the heterogeneity of immune cell populations and their necroptosis gene expression profiles, offering valuable insights into the immune landscape of sepsis. Our findings suggest that these genes may contribute to the dysregulated immune response observed in sepsis, potentially serving as new targets for therapeutic intervention. By integrating multi-omics data and advanced bioinformatics techniques, this study sheds light on molecular alterations in sepsis, paving the way for improved diagnostic and therapeutic strategies (Tang et al., 2019).

Single-cell data analysis revealed that six necroptotic genes were differentially expressed in immune cells. These genes are *CTSS*, *TXN*, *MYH9*, *FPR1*, *FMR1*, and *MPRIP*. Among these, *CTSS* was highly expressed in various types of immune cells. *CTSS*, also known as Cathepsin S, is a cysteine protease that degrades proteins within lysosomes. It is predominantly expressed in immune cells, such as professional antigen-presenting cells (APCs), B cells, dendritic cells (DCs) and macrophages (Riese and Chapman, 2000). With predominant expression in both DCs and B cells, which are professional APCs, *CTSS* plays an important role in the immune response. Specifically, *CTSS* activity fundamentally drives MHC class II maturation, antigen processing and presentation (Smyth et al., 2022). In the context of sepsis, *CTSS* has been implicated in modulating immune responses and inflammation. The increased expression of *CTSS* might contribute to the excessive inflammation and tissue damage in sepsis. It promotes the degradation of extracellular matrix components and facilitating the infiltration of immune cells into tissues, —both of which are hallmarks of sepsis (Turk et al., 2012). *CTSS* involvement in inflammation is also evident in necroptosis, a form of programmed cell death that plays an intrinsic role in inflammatory processes (Dhuriya and Sharma, 2018). *CTSS* has been shown to cleave RIP1 kinase, which limiting necroptosis within macrophages. Therefore, inhibiting *CTSS* along with caspase-8 may offer a promising treatment strategy for various inflammatory conditions (McComb et al., 2014). This is consistent with our findings that *CTSS* is one of the key DEGs in sepsis; however, this is the first time it has been found to play a role in the necroptotic process of sepsis.

Thioredoxin (TXN) is a small redox protein. It plays a pivotal role in maintaining cellular redox homeostasis by reducing oxidized proteins. And it is involved in various cellular processes, such as DNA synthesis, repair, and apoptosis (Holmgren, 1985). In sepsis, *TXN* exerts protective effects by mitigating oxidative stress and modulating inflammatory responses (Nakamura et al., 1997). Previous studies have suggested that *TXN* is a key regulator in maintaining endoplasmic reticulum (ER) homeostasis in sepsis. Additionally, it has been identified as a diagnostic biomarker strongly associated with immune cell infiltration in sepsis (Zhou et al., 2023b). Building on these findings, our results indicate that *TXN* is highly expressed in CD4^+^ T cells, CD8^+^ T cells, monocytes, neutrophils, and natural killer (NK) cells. The upregulation of *TXN* during sepsis may represent a compensatory mechanism to counteract excessive oxidative stress and inflammation.

Myosin Heavy Chain 9 (MYH9) is a non-muscle myosin that participates in several cellular processes such as cell motility, adhesion, and cytokinesis (Conti and Adelstein, 2008). Recent studies have suggested that *MYH9* plays a role in immune function and inflammatory responses (Chung et al., 2019). In sepsis, *MYH9* expression has been linked to altered immune cell dynamics and impaired immune responses. Our study identified significantly elevated expression of *MYH9* in CD4^+^ T cells, CD8^+^ T cells, and HSC, as well as in monocytes, neutrophils and NK cells, indicating its potential involvement in the immune dysregulation observed in sepsis. The mechanisms by which *MYH9* contributes to sepsis pathophysiology remain unclear. Furthermore, its role in cytoskeletal organization and cell signaling suggests it could be a critical mediator of immune cell function in the context of septic.

Formyl Peptide Receptor 1 (FPR1) is a member of the human G protein-coupled receptor (GPCR) family in humans and plays a key role in mediating the chemotaxis of immune cells, particularly neutrophils and macrophages, to sites of infection and inflammation (Le et al., 2002). *FPR1* is crucial for host defense, regulating the migration and activation of immune cells in response to bacterial peptides (Ye and Boulay, 1997). Previous studies have demonstrated that *FPR1* is vital in modulating the innate immune response through neutrophils (Lee et al., 2018), and elevated *FPR1* expression contribute to excessive neutrophil infiltration (Chung et al., 2008). Recent reports indicate that *FPR1* is also expressed in adaptive immune cells, in addition to innate immune cells involved in inflammation (Nagaya et al., 2017). Notably, *FPR1* is expressed in the nuclei of naïve CD4 T cells and regulates their chemotactic migration (Lee et al., 2018). In the context of sepsis, dysregulated *FPR1* expression leads to impaired immune cell recruitment and function; consequently, *FPR1* has been suggested as a putative target for several diseases, including sepsis (Lee et al., 2017). Our findings show higher *FPR1* expression in monocytes and neutrophils, which consistent with previous literature, but no significant difference was found in the expression levels in CD4^+^ T cells between SE and HC groups.

The Fragile X Mental Retardation 1 (FMR1) gene encodes the Fragile X Mental Retardation Protein (FMRP), which participates in synaptic function and neuronal development (Bagni and Greenough, 2005). Although primarily studied in neurological disorders, recent research indicates that *FMR1* also play a role in immune regulation (Bhakar et al., 2012). In sepsis, *FMR1* expression has been associated with altered immune cell function and inflammatory responses; moreover, the absence of FMRP induces TNF-mediated apoptosis and necroptosis in infections and liver diseases (Zhuang et al., 2020). Additionally, *FMR1* has been shown to play a role in the stimulus-induced localization of mRNAs in dendritic cells (Dictenberg et al., 2008). Building on this, our findings of significantly higher *FMR1* expression in CD8^+^ T cells suggest its contribution to the necroptosis in sepsis, warranting further investigation of the underlying mechanisms.

GO analysis revealed that these DEGs are prominently involved in regulation of apoptotic signaling pathways and the IκB kinase/NF-κB signaling pathway, both of which are critical for cell death, inflammation, and immune response modulation (Xu and Shi, 2007). Additionally, KEGG pathway enrichment analysis further highlighted the involvement of these DEGs in programmed cell death pathways, including necroptosis and apoptosis, as well as the NOD-like receptor signaling pathway, underscoring their pivotal roles in innate immunity (Zhou et al., 2024).

Necroptosis is a form of programmed cell death distinct from apoptosis and is inflammatory, potentially exacerbating the immune response in sepsis (Liu et al., 2021). The enrichment of DEGs in the necroptosis pathway suggests that this form of cell death contributes significantly to the pathogenesis of sepsis by promoting inflammation and tissue damage (Pasparakis and Vandenabeele, 2015). Similarly, the NOD-like receptor signaling pathway is essential for in recognizing pathogens and initiating immune responses in sepsis (Zhang and Ning, 2021). The significant enrichment of DEGs in the NOD-like receptor signaling suggests a potential mechanism through which sepsis may induce a robust immune response, leading to systemic inflammation and organ dysfunction (Foley et al., 2015).

Our GSVA revealed notable differences between the HC and SE groups. Pathways such as oxidative phosphorylation, sphingolipid metabolism and glycosphingolipid biosynthesis (lacto and neolacto series) were downregulated in the SE group. Conversely, the Wnt signaling pathway was upregulated. Oxidative phosphorylation is essential for ATP production and the cellular energy metabolism. Consequently, the downregulation of oxidative phosphorylation may reflect mitochondrial dysfunction commonly observed in sepsis (Nedel et al., 2023). The analysis of the sphingolipid metabolism pathway revealed that plasma levels of plasmalogens, circulating antioxidant phospholipids, decreased in sepsis patients. These plasmalogens provide critical protection against oxidative insults by preserving cellular lipid integrity during stress (Chang et al., 2023). Similarly, the glycosphingolipid biosynthesis pathway (lacto and neolacto series) showed significant downregulation in sepsis. This finding therefore further supports the notion that sphingolipid metabolic reprogramming is a hallmark of this condition (Li et al., 2025).

The Wnt signaling pathway, known for regulating cell proliferation and differentiation, is upregulated and might indicate a compensatory mechanism or a shift in cellular dynamics in response to sepsis (Teo and Kahn, 2010). The Wnt signaling pathway is an evolutionarily conserved system that is essential for organ development and adult homeostasis. This pathway regulates proliferation, differentiation, apoptosis, motility, and polarization of cells (Schaale et al., 2011). Wnt signaling is classified into canonical (β-catenin-dependent) and non-canonical (β-catenin-independent) pathways, with the canonical pathway capable of regulating anti-inflammatory responses (Schaale et al., 2011). Recent evidence identifies Wnt proteins and signaling components as key modulators of the immune system, involved in balancing between tolerance and immunity. DCs have been identified as direct targets of this regulation (Zhou et al., 2009; Manicassamy et al., 2010). The Wnt signaling pathway may coordinate the interaction between the body’s innate and adaptive immunity in response to infection (Brandenburg and Reiling, 2016). A recently published study showed that inhibition of Wnt/β-catenin signaling reduces inflammation and mitigates sepsis-induced organ injury in experimental models (Sharma et al., 2017). Based on these findings, targeting the Wnt/β-catenin pathway may provide a potential therapeutic approach for the treatment of sepsis.

These findings suggest metabolic dysregulation and altered signaling pathways in sepsis, which may contribute to the disease’s complex pathophysiology by disrupting cellular homeostasis and immune responses (Wasyluk and Zwolak, 2021). Additionally, GSEA further confirmed the enrichment of DEGs in pathways such as G alpha (s) signaling pathway and olfactory transduction. The G alpha (s) signaling pathway participates in various cellular processes, including hormone signaling and metabolic regulation. Disruption of this pathway could play a significant role in sepsis progression (Prossnitz and Barton, 2023). While olfactory transduction is primarily associated with the sense of smell, it has been implicated in immune responses and inflammation, suggesting a broader role in sepsis pathophysiology (Shepard and Pluznick, 2016).

Our ssGSEA revealed significant differences in immune cell infiltration between SE and HC groups, particularly in activated B cells and CD4^+^ T cells, indicating an imbalance that may drive the exaggerated inflammatory response and immune dysregulation in sepsis (Liu et al., 2022) and may be associated with the identified DEGs (Xue et al., 2023). Single-cell RNA sequencing allowed us to detail the cellular composition and revealed neutrophils as the predominant cell type in sepsis. This finding supports the established role of neutrophils in driving the inflammatory response during sepsis (Hong et al., 2022).

The initial inflammatory response to sepsis is primarily driven by innate immune cells in the immune system, including neutrophils, monocytes, and macrophages. These cells can release a variety of inflammatory cytokines (Muszynski et al., 2016). Neutrophils play a crucial role in sepsis-induced inflammation and immune imbalance, serving as the host’s first line of defense against infections (van der Poll et al., 2017). While neutrophils exhibit a powerful antimicrobial effect, they also have a double-edged role, serving as both vital guardians of host defenses and harmful facilitators of tissue damage in an uncontrolled inflammatory state (Liew and Kubes, 2019). The dual role of SAP has recently gained new validation; in Gram-negative bacterial pneumonic sepsis, the adaptor protein SAP enhances host defense by promoting neutrophil extracellular trap (NET) formation. SAP-deficient mice showed impaired NET generation, which led to compromised bacterial clearance and consequently significantly exacerbated lung damage and systemic infection (Tripathi et al., 2019). In septic patients, dysregulated neutrophil cell death can be detrimental, as it leads to immune-related organ failure, reducing host defenses and increasing susceptibility to hospital-acquired infections. Consequently, neutrophils shift from being potent antibacterial agents to harmful mediators that cause tissue damage and organ failure. Under septic conditions, spontaneous apoptosis of neutrophils is inhibited, resulting in increased longevity of these cells (Wang et al., 2021). The delayed apoptosis of neutrophils in sepsis is a significant causative factor in septic organ dysfunction. Research has demonstrated that anti-PD-L1 treatment can improve survival in septic mice (Wang et al., 2015), suggesting that promoting apoptosis in septic neutrophils may represent an effective therapeutic target for the treatment of sepsis. It is noteworthy that apoptosis is not the sole critical cell death pathway; neutrophil extracellular trap (NET) formation also serves as an essential antibacterial mechanism. Deficiency in the regulatory protein SAP impedes the release of DNA–antimicrobial protein complexes by neutrophils. Adoptive transfer experiments further reveal that SAP regulates NETosis indirectly, through mechanisms independent of neutrophils, unveiling novel therapeutic avenues for immunomodulation (Tripathi et al., 2019). In 2021, new developments emerged regarding disulfiram, an FDA-approved drug repurposed as a targeted inhibitor of neutrophil extracellular traps (NETs) and pyroptosis. This drug inhibits septic neutrophil gasdermin D (*GSDMD*) activation and reduces NET release, thereby improving sepsis-induced organ damage and survival in septic mice (Silva et al., 2021). This discovery, together with SAP research (Tripathi et al., 2019), clarifies that simultaneously targeting distinct cell death pathways—apoptosis, pyroptosis, and neutrophil extracellular trap (NET) formation—and their regulatory factors, such as PD-L1, GSDMD, and SAP, constitutes a synergistic therapeutic strategy for sepsis. Notably, as the first identified positive regulatory adaptor promoting NET formation, SAP facilitates extracellular entrapment mechanisms that enable innovative approaches to overcome immunoparalysis, a state of immune suppression commonly observed in sepsis. In summary, targeting neutrophil cell death may be a promising therapeutic strategy against sepsis.

Our study provides significant insights; however, it also has inherent limitations that should be considered. We identified differential expression patterns of NRGs in sepsis and their profound impact on immune dysregulation. However, these findings require validation in large-scale prospective cohorts using external datasets before clinical translation. We used ssGSEA, because of its sensitivity in quantifying various immune cell subsets and wide applicability. However, cross-validation with alternative algorithms (e.g., quanTIseq, xCell) was precluded by limitations in access to compatible raw data formats and computational resources. This highlights the need for future studies with larger sample sizes to validate immune infiltration patterns using integrated computational methods. Our transcriptomic analyses revealed correlations between NRGs and immune responses. However, establishing causal mechanisms requires further *in vitro* and *in vivo* experimental validation, especially to understand how NRGs regulate immune cell function and inflammatory cascades.

## Conclusion

Overall, our findings provide a comprehensive understanding of the molecular mechanisms underlying sepsis, especially the critical role of necroptosis-related genes in immune responses, which underscore potential targets for therapeutic intervention. Further research is needed to explore these pathways in detail and to confirm their clinical relevance for sepsis management.

## Data Availability

The datasets presented in this study can be found in online repositories. The names of the repository/repositories and accession number(s) can be found in the article/**Supplementary Material**.
