# Supplementary figures and images for: The relationship between immune cell infiltration and necroptosis gene expression in sepsis: an analysis using single-cell transcriptomic data
Source: Front Cell Infect Microbiol. 2025 Aug 11;15:1618438. doi: 10.3389/fcimb.2025.1618438 (PMC12375562; doi:10.3389/fcimb.2025.1618438)

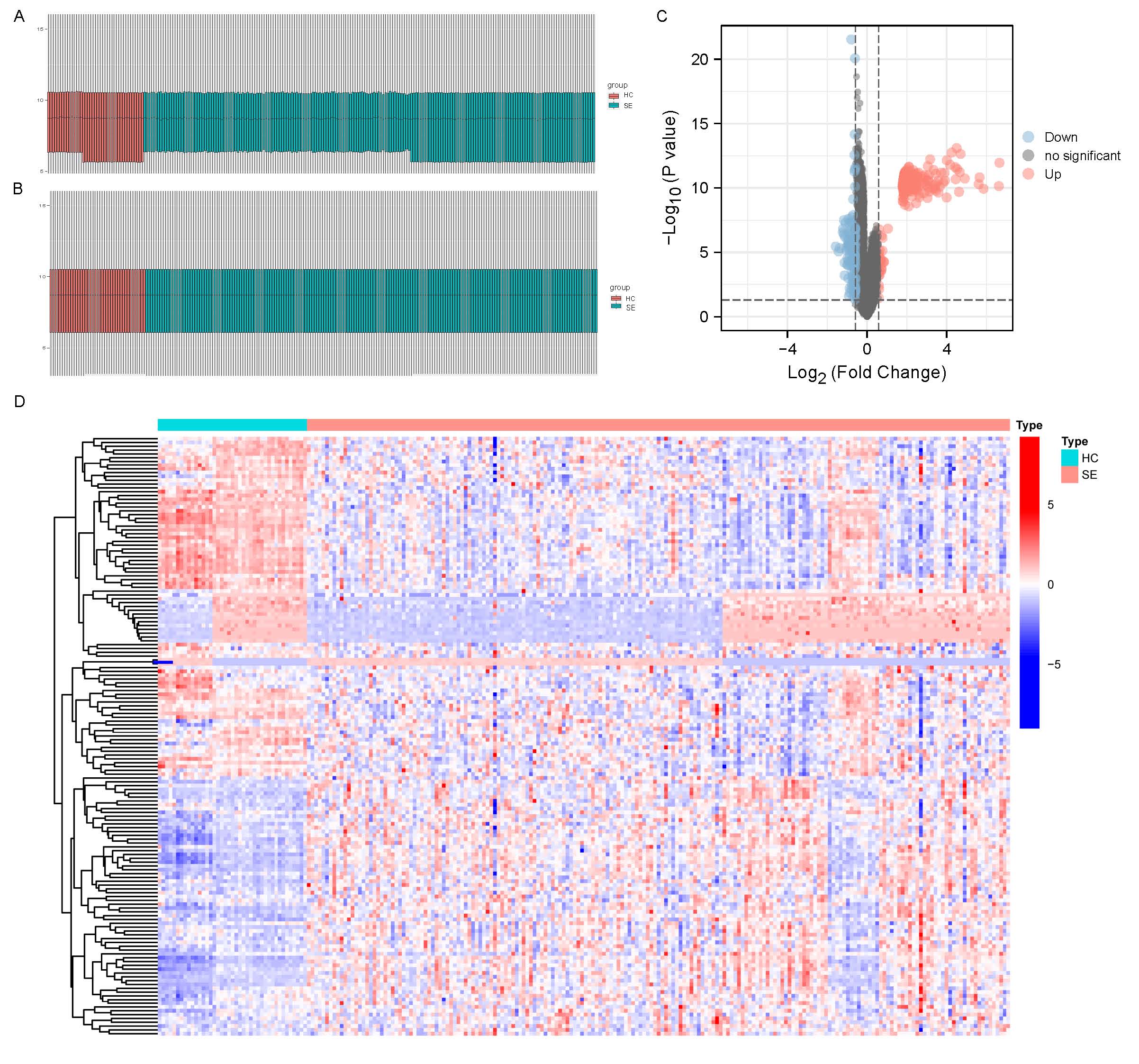

Supplement: Supplementary file 1 [file Image1.jpeg]

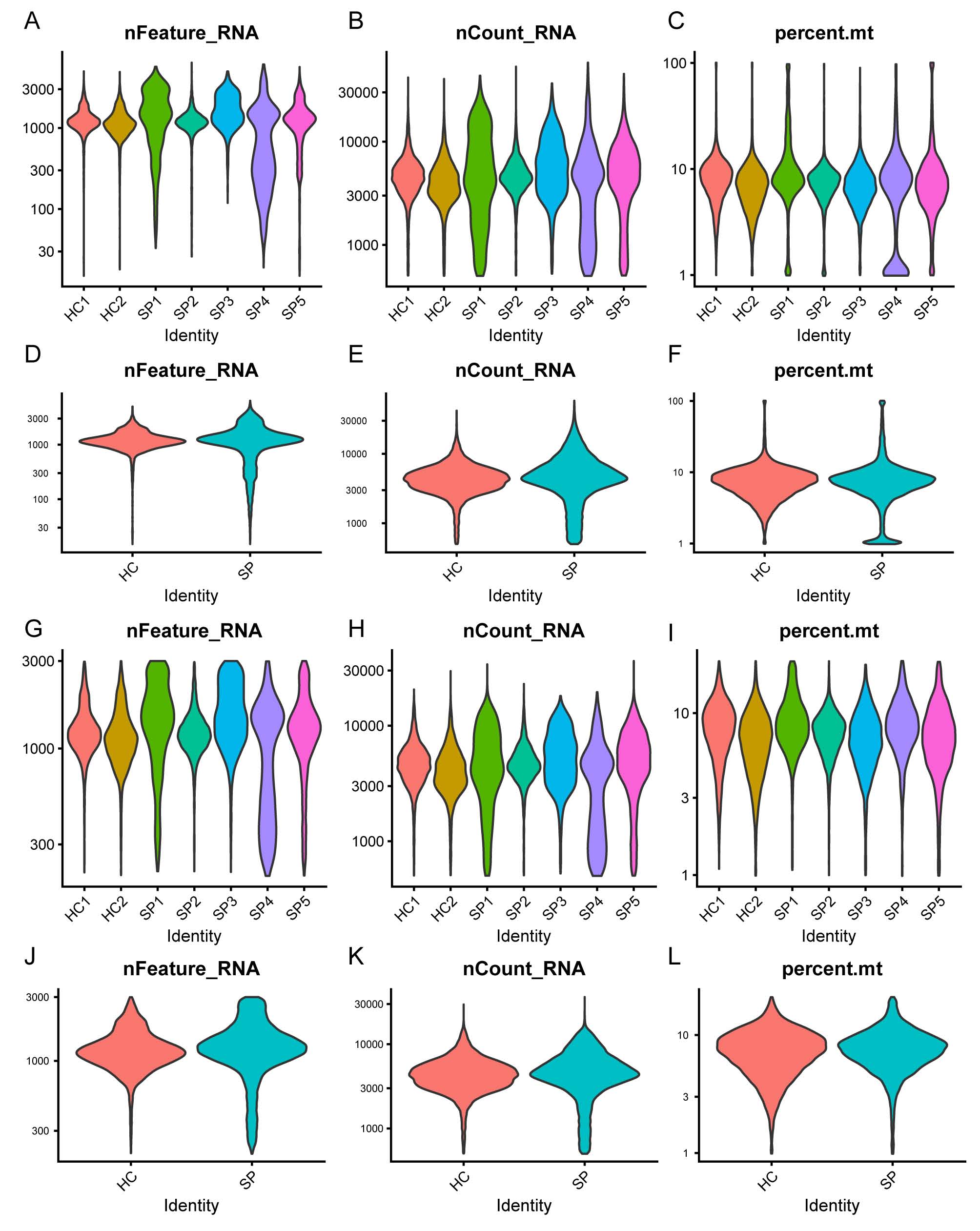

Supplement: Supplementary file 2 [file Image2.jpeg]

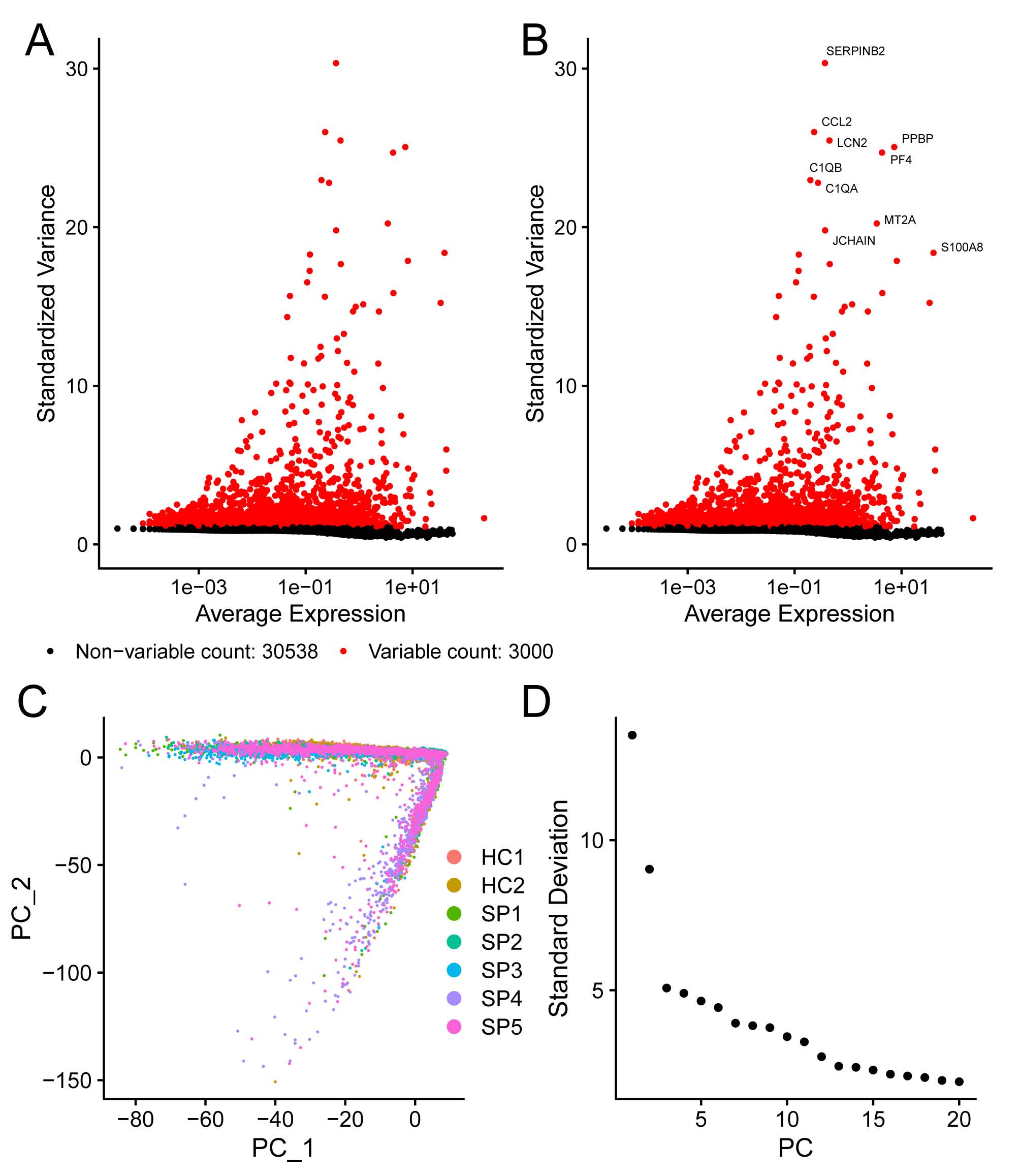

Supplement: Supplementary file 3 [file Image3.jpeg]
