# Supplementary material for: The relationship between immune cell infiltration and necroptosis gene expression in sepsis: an analysis using single-cell transcriptomic data
Source: Front Cell Infect Microbiol. 2025 Aug 11;15:1618438. doi: 10.3389/fcimb.2025.1618438 (PMC12375562; doi:10.3389/fcimb.2025.1618438)
Supplement: Supplementary file 4 [file Table1.docx]

**Table 1. GEO Dataset Information of Sepsis Patient Sample Source**

| ID | GEO number | Platforms | Sample | Source types | Reference |
| --- | --- | --- | --- | --- | --- |
| 1 | GSE131761 | GPL13497 | 15HC, 114SE | PBMC | P. Martínez-Paz, M et.al[3] |
| 2 | GSE49541 | GPL20301 | 26HC  79SE | PBMC | V. Herwanto, B. et.al[4] |

**Table 2. Analysis of GO enrichment of differentially expressed necrotic apoptosis gene**

| ONTOLOGY | ID | Description | GeneRatio | BgRatio | P value | p. adjust | Q value |
| --- | --- | --- | --- | --- | --- | --- | --- |
| BP | GO:2001233 | regulation of apoptotic signaling pathway | 36/156 | 406/18670 | 8.40e-27 | 3.34e-23 | 1.84e-23 |
| BP | GO:0097300 | programmed necrotic cell death | 18/156 | 49/18670 | 1.33e-25 | 2.65e-22 | 1.45e-22 |
| BP | GO:0070265 | necrotic cell death | 19/156 | 62/18670 | 3.36e-25 | 4.45e-22 | 2.45e-22 |
| BP | GO:0070266 | necroptotic process | 17/156 | 44/18670 | 1.09e-24 | 1.08e-21 | 5.96e-22 |
| BP | GO:0007249 | I-kappaB kinase/NF-kappaB signaling | 28/156 | 269/18670 | 8.33e-23 | 6.63e-20 | 3.64e-20 |
| CC | GO:0035631 | CD40 receptor complex | 6/157 | 11/19717 | 1.04e-10 | 4.02e-08 | 2.90e-08 |
| CC | GO:0045121 | membrane raft | 16/157 | 315/19717 | 4.78e-09 | 6.47e-07 | 4.67e-07 |
| CC | GO:0098857 | membrane microdomain | 16/157 | 316/19717 | 5.00e-09 | 6.47e-07 | 4.67e-07 |
| CC | GO:0098589 | membrane region | 16/157 | 328/19717 | 8.49e-09 | 8.24e-07 | 5.94e-07 |
| CC | GO:0098562 | cytoplasmic side of membrane | 11/157 | 178/19717 | 1.90e-07 | 1.43e-05 | 1.03e-05 |
| MF | GO:0032813 | tumor necrosis factor receptor superfamily binding | 10/154 | 46/17697 | 5.78e-12 | 2.73e-09 | 2.15e-09 |
| MF | GO:0005126 | cytokine receptor binding | 18/154 | 286/17697 | 6.08e-11 | 1.21e-08 | 9.47e-09 |
| MF | GO:0031625 | ubiquitin protein ligase binding | 18/154 | 290/17697 | 7.64e-11 | 1.21e-08 | 9.47e-09 |
| MF | GO:0005164 | tumor necrosis factor receptor binding | 8/154 | 31/17697 | 1.82e-10 | 1.85e-08 | 1.45e-08 |
| MF | GO:0044389 | ubiquitin-like protein ligase binding | 18/154 | 308/17697 | 2.04e-10 | 1.85e-08 | 1.45e-08 |

（biological process， BP，molecular function， MF, cellular component， CC）

**Table 3. KEGG enrichment analysis of differentially expressed** **Necroptosis genes**

| ONTOLOGY | ID | Description | GeneRatio | BgRatio | P value | p. adjust | Q value |
| --- | --- | --- | --- | --- | --- | --- | --- |
| KEGG | hsa04217 | Necroptosis | 34/124 | 159/8076 | 2.08e-30 | 4.20e-28 | 2.08e-28 |
| KEGG | hsa04621 | NOD-like receptor signaling pathway | 32/124 | 181/8076 | 8.16e-26 | 8.24e-24 | 4.08e-24 |
| KEGG | hsa04210 | Apoptosis | 27/124 | 136/8076 | 3.39e-23 | 2.28e-21 | 1.13e-21 |
| KEGG | hsa04668 | TNF signaling pathway | 21/124 | 112/8076 | 1.29e-17 | 6.52e-16 | 3.23e-16 |
| KEGG | hsa04064 | NF-kappa B signaling pathway | 19/124 | 104/8076 | 8.56e-16 | 3.46e-14 | 1.71e-14 |

KEGG（Kyoto Encyclopedia of Genes and Genomes

**Table 4. GSEA Analysis of Differentially Expressed Necroptosis Gene**

| Description | Enrichment Score | NES | P value | P  adjust | Q  values |
| --- | --- | --- | --- | --- | --- |
| REACTOME_G_ALPHA_S_SIGNALLING_EVENTS | 0.770211446 | 2.358059 | 0.001003 | 0.165169 | 0.160636 |
| KEGG_OLFACTORY_TRANSDUCTION | 0.812289541 | 2.489814 | 0.001007 | 0.165169 | 0.160636 |
| REACTOME_OLFACTORY_SIGNALING_PATHWAY | 0.824059728 | 2.519941 | 0.00101 | 0.165169 | 0.160636 |
| REACTOME_TRANSLATION | 0.59943358 | 1.826815 | 0.001016 | 0.165169 | 0.160636 |
| REACTOME_KERATINIZATION | 0.776975342 | 2.32879 | 0.001056 | 0.165169 | 0.160636 |
| REACTOME_RRNA_PROCESSING | 0.646084133 | 1.920877 | 0.001059 | 0.165169 | 0.160636 |
| REACTOME_INFLUENZA_INFECTION | 0.633488604 | 1.856762 | 0.001086 | 0.165169 | 0.160636 |
| REACTOME_FORMATION_OF_THE_CORNIFIED_ENVELOPE | 0.678227726 | 1.970082 | 0.001104 | 0.165169 | 0.160636 |
| REACTOME_EUKARYOTIC_TRANSLATION_INITIATION | 0.712587912 | 2.044772 | 0.001124 | 0.165169 | 0.160636 |
| REACTOME_NONSENSE_MEDIATED_DECAY_NMD_ | 0.675342143 | 1.936211 | 0.001124 | 0.165169 | 0.16063t6 |
| REACTOME_SRP_DEPENDENT_COTRANSLATIONAL_PROTEIN_TARGETING_TO_MEMBRANE | 0.631036631 | 1.805095 | 0.001133 | 0.165169 | 0.160636 |
| REACTOME_SELENOAMINO_ACID_METABOLISM | 0.604724236 | 1.722116 | 0.001142 | 0.165169 | 0.160636 |
| REACTOME_RESPONSE_OF_EIF2AK4_GCN2_TO_AMINO_ACID_DEFICIENCY | 0.674986639 | 1.906874 | 0.001164 | 0.165169 | 0.160636 |
| REACTOME_EUKARYOTIC_TRANSLATION_ELONGATION | 0.737384261 | 2.064597 | 0.001174 | 0.165169 | 0.160636 |
| WP_CYTOPLASMIC_RIBOSOMAL_PROTEINS | 0.71832775 | 1.994996 | 0.001189 | 0.165169 | 0.160636 |
| KEGG_RIBOSOME | 0.696753684 | 1.933084 | 0.001193 | 0.165169 | 0.160636 |
| REACTOME_ANTIMICROBIAL_PEPTIDES | 0.70943368 | 1.966167 | 0.001193 | 0.165169 | 0.160636 |
| REACTOME_ACTIVATION_OF_THE_MRNA_UPON_BINDING_OF_THE_CAP_BINDING_COMPLEX_AND_EIFS_AND_SUBSEQUENT_BINDING_TO_43S | 0.733858955 | 1.923958 | 0.001297 | 0.166643 | 0.162069 |
| REACTOME_DEFENSINS | 0.849945284 | 2.14533 | 0.001376 | 0.166643 | 0.162069 |
| REACTOME_BETA_DEFENSINS | 0.893766352 | 2.186917 | 0.001416 | 0.166643 | 0.162069 |

**Table 5. GSVA of Differentially Expressed Necroptosis Gene**

| ID | logFC | AveExpr | t | P  Value | adj.P.Val |
| --- | --- | --- | --- | --- | --- |
| KEGG_FOLATE_BIOSYNTHESIS | 0.577841121 | -0.012151684 | 14.38643027 | 2.69E-34 | 4.95E-32 |
| KEGG_PPAR_SIGNALING_PATHWAY | 0.279802276 | 0.001325235 | 12.37405325 | 1.48E-27 | 1.36E-25 |
| KEGG_ALZHEIMERS_DISEASE | 0.37381006 | -0.000674165 | 11.71100641 | 2.24E-25 | 1.37E-23 |
| KEGG_GALACTOSE_METABOLISM | 0.415725209 | 0.003198806 | 11.48354235 | 1.23E-24 | 5.03E-23 |
| KEGG_GLUTATHIONE_METABOLISM | 0.370157879 | 0.004937705 | 11.4696631 | 1.37E-24 | 5.03E-23 |
| KEGG_COMPLEMENT_AND_COAGULATION_CASCADES | 0.325597464 | -0.01317731 | 11.02094352 | 3.84E-23 | 1.18E-21 |
| KEGG_OXIDATIVE_PHOSPHORYLATION | 0.504463832 | 0.001486321 | 10.67567731 | 4.86E-22 | 1.28E-20 |
| KEGG_WNT_SIGNALING_PATHWAY | -0.232453135 | 0.005746821 | -10.503292 | 1.71E-21 | 3.76E-20 |
| KEGG_T_CELL_RECEPTOR_SIGNALING_PATHWAY | -0.358397783 | 0.014994228 | -10.49317867 | 1.84E-21 | 3.76E-20 |
| KEGG_SNARE_INTERACTIONS_IN_VESICULAR_TRANSPORT | 0.392969864 | -0.004999827 | 10.35112689 | 5.15E-21 | 9.31E-20 |
| KEGG_VIRAL_MYOCARDITIS | -0.358017635 | 0.004327859 | -10.34038274 | 5.56E-21 | 9.31E-20 |
| KEGG_CIRCADIAN_RHYTHM_MAMMAL | -0.521823834 | 0.021349867 | -10.29133732 | 7.93E-21 | 1.22E-19 |
| KEGG_GLYCOSPHINGOLIPID_BIOSYNTHESIS_LACTO_AND_NEOLACTO_SERIES | 0.342082971 | 0.019490901 | 10.0398858 | 4.83E-20 | 6.83E-19 |
| KEGG_CARDIAC_MUSCLE_CONTRACTION | 0.301853489 | -0.003954726 | 9.929606872 | 1.06E-19 | 1.39E-18 |
| KEGG_DRUG_METABOLISM_OTHER_ENZYMES | 0.286915292 | -0.011261668 | 9.743832044 | 3.96E-19 | 4.85E-18 |
| KEGG_PEROXISOME | 0.266754841 | 0.002166357 | 9.609397546 | 1.02E-18 | 1.17E-17 |
| KEGG_AMINO_SUGAR_AND_NUCLEOTIDE_SUGAR_METABOLISM | 0.354666537 | 0.002318951 | 9.529619786 | 1.78E-18 | 1.93E-17 |
| KEGG_HUNTINGTONS_DISEASE | 0.314017001 | -0.002637526 | 9.241877111 | 1.32E-17 | 1.35E-16 |
| KEGG_SPHINGOLIPID_METABOLISM | 0.252943244 | 0.010408938 | 9.060988244 | 4.57E-17 | 4.42E-16 |
| KEGG_PARKINSONS_DISEASE | 0.430009813 | -0.003477008 | 8.986104567 | 7.61E-17 | 7.00E-16 |

**Figure captions**

**Figure S1. Differentially expressed gene analysis in combined datasets**

A. the distribution of between-sample expression profiles prior to combination and correction of the two datasets.

B. the distribution of between-sample expression profiles after combination and correction of the two datasets.

C. a volcano plot was constructed based on differential genetic analysis, with log2 values plotted on the abscissa and with -log10 values plotted on the ordinate. Red nodes indicate up-regulated differentially expressed genes, blue nodes represent down-regulated differentially expressed genes, and black nodes indicate genes that are not significantly differentially expressed.

D. A heat map was generated to visualize the differential expression of genes between the SE (Sepsis)group and the HC (Health control) group, with red indicating up-regulation and blue indicating down-regulation of expression.

**Figure S2. Cellular quality control of single-cell sequencing data**

(A) Violin plot of the number of genes for each sample before QC filtration；

(B) Violin plot of the nCount number of each sample before QC filtration；

(C) Proportion of mitochondrial genes in each sample before QC filtration;

(D) The number of genes in the SP and HC groups before quality control filtration;

(E) The number of nCounts for the SP and HC groups before QC filtration;

(F) The proportion of mitochondrial genes in the SP group and HC group before quality control filtration;

(G) violin plot of the number of genes in each sample after QC filtering;

(H) violin plot of the nCount number of each sample after quality control filtering;

(I) the proportion of mitochondrial genes in each sample after quality control filtering;

(J) The number of genes in the SP group and HC group after quality control and filtration;

(K) the number of nCounts in the SP group and HC group after QC filtering;

(L) The proportion of mitochondrial genes in the SP group and the HC group after quality control filtration;

(HC, Health control; SP, Sepsis Patients; UMI, unique molecular identifiers)

**Figure S3. Optimal threshold screening for dimensionality reduction clustering of single-cell sequencing data.**

(A) The standard deviation scatter plot shows the hypervariable genes present in the cell;

(B) The standard deviation scatter plot shows the hypervariable top 10 genes.

(C) PC analysis distribution of samples from SP group and control group.

(D) The best principal component screening plot was selected as 17 PCs with smooth curves.

(PC, principal component; SP, Sepsis Patients)

**Gene Abbreviation Index**

C1QA - Complement C1q A Chain

C1QB - Complement C1q B Chain

CCL2 - C-C Motif Chemokine Ligand 2 (MCP-1)

CD177 - CD177 Molecule

CDK9 - Cyclin Dependent Kinase 9

CEBPB - CCAAT Enhancer Binding Protein Beta

CXCL8 - C-X-C Motif Chemokine Ligand 8 (IL-8)

HTRA2 - HtrA Serine Peptidase 2

JCHAIN - Joining Chain of Multimeric IgA and IgM

LCN2 - Lipocalin 2

LTB - Lymphotoxin Beta

LTF - Lactotransferrin

MAP1LC3B - Microtubule Associated Protein 1 Light Chain 3 Beta

MAPK8 - Mitogen-Activated Protein Kinase 8 (JNK1)

MCL1 - MCL1 Apoptosis Regulator, BCL2 Family Member

MMP9 - Matrix Metallopeptidase 9

MT2A - Metallothionein 2A

MTX1 - Metaxin 1

PF4 - Platelet Factor 4 (CXCL4)

PGLYRP1 - Peptidoglycan Recognition Protein 1

PPBP - Pro-Platelet Basic Protein (CXCL7)

RPL10 - Ribosomal Protein L10

RPL13 - Ribosomal Protein L13

RPL28 - Ribosomal Protein L28

RPLP2 - Ribosomal Protein Lateral Stalk Subunit P2

RPS6 - Ribosomal Protein S6

RPS27 - Ribosomal Protein S27

S100A8 - S100 Calcium Binding Protein A8

S100A12 - S100 Calcium Binding Protein A12

SAT1 - Spermidine/Spermine N1-Acetyltransferase 1

SERPINB2 - Serpin Family B Member 2

USP22 - Ubiquitin Specific Peptidase 22
